# Quantum non-demolition measurement of a many-body Hamiltonian

**DOI:** 10.1038/s41467-020-14489-5

**Published:** 2020-02-07

**Authors:** Dayou Yang, Andrey Grankin, Lukas M. Sieberer, Denis V. Vasilyev, Peter Zoller

**Affiliations:** 1 Center for Quantum Physics, University of Innsbruck, 6020 Innsbruck, Austria; 2 Institute for Quantum Optics and Quantum Information of the Austrian Academy of Sciences, 6020 Innsbruck, Austria

**Keywords:** Quantum simulation, Theoretical physics

## Abstract

In an ideal quantum measurement, the wave function of a quantum system collapses to an eigenstate of the measured observable, and the corresponding eigenvalue determines the measurement outcome. If the observable commutes with the system Hamiltonian, repeated measurements yield the same result and thus minimally disturb the system. Seminal quantum optics experiments have achieved such quantum non-demolition (QND) measurements of systems with few degrees of freedom. In contrast, here we describe how the QND measurement of a complex many-body observable, the Hamiltonian of an interacting many-body system, can be implemented in a trapped-ion analog quantum simulator. Through a single-shot measurement, the many-body system is prepared in a narrow band of (highly excited) energy eigenstates, and potentially even a single eigenstate. Our QND scheme, which can be carried over to other platforms of quantum simulation, provides a framework to investigate experimentally fundamental aspects of equilibrium and non-equilibrium statistical physics including the eigenstate thermalization hypothesis and quantum fluctuation relations.

## Abstract (web summary)

The theoretical understanding of quantum many-body systems often involves properties of energy eigenstates but these are difficult to probe experimentally. Yang et al. propose an experiment that supports preparation and measurement of single eigenstates, enabling detailed studies of statistical physics.

## Introduction

Recent experimental advances provide intriguing opportunities in the preparation, manipulation, and measurement of the quantum state of engineered complex many-body systems. This includes the ability to address individual sites of lattice systems enabling single-shot read-out of single-particle observables, as demonstrated by the quantum gas microscope for atoms in optical lattices^1,2^, single-spin or qubit read-out of trapped ions^3–7^ and Rydberg tweezers arrays^8–12^, and superconducting qubits^13,14^. In contrast, we are interested below in developing single-shot measurements of many-body observables such as the Hamiltonian $\hat{H}$ of an interacting many-body system. For an isolated quantum system, $\hat{H}$ represents a quantum non-demolition (QND) observable, and our goal is to implement a QND measurement of energy of a quantum many-body system in an analog simulator setting. We note that quantum optics provides with several examples of QND measurements; however these have so far been confined to observables representing few quantum degrees of freedom^14–20^.

Developing QND measurement of a many-body Hamiltonian $\hat{H}$ provides us with the unique opportunity to distill—in a single run of the experiment—an energy eigenstate $\left|\ell \right\rangle$ from an initial, possibly mixed, or finite temperature state, by observing in particular run the energy eigenvalue *E*_*ℓ*_. In case of measurement with finite resolution, this will prepare states in a narrow energy window, reminiscent of a microcanonical ensemble. We emphasize that state preparation by measurement is intrinsically probabilistic, that is, will vary from shot to shot, reflecting the population distribution. Furthermore, this provides us with a tool to determine populations and population distributions of (excited) energy eigenstates, as required in, for example, many-body spectroscopy^21^. The ability to prepare and measure (single) energy eigenstates provides us with a unique tool to address experimentally fundamental problems in quantum statistical physics, such as the eigenstate thermalization hypothesis (ETH)^22–24^, which asserts that single energy eigenstates of an (isolated) ergodic system encode thermodynamic equilibrium properties. Developing the capability to turn QND measurements on and off allows one to alternate between time periods of free evolution of the unobserved many-body quantum system and energy measurement. This allows quantum feedback in a many-body system conditional to measurement outcomes, and in particular provides a framework to monitor non-equilibrium dynamics and processes in quantum thermodynamics^25^, including measurement of work functions and quantum fluctuation relations (QFRs)^26–28^. These relations express fundamental constraints on, for example, the work performed on a quantum system in an arbitrary non-equilibrium process, imposed by the universal canonical form of thermal states and the principle of microreversibility.

Our aim below is to develop QND measurement of $\hat{H}$ in physical settings of analog quantum simulation, in particular exploring the regime of mesoscopic system sizes. We will demonstrate this in detail as an example of an analog trapped-ion quantum simulator, realizing a long-range transverse Ising Hamiltonian and the associated QND measurement. Our implementation in an analog quantum device should be contrasted to QND measurement of $\hat{H}$ via a phase estimation algorithm^29^, which however requires a universal (digital) quantum computer.

## Results

### QND measurement of $\hat{H}$

**Fig. 1:**
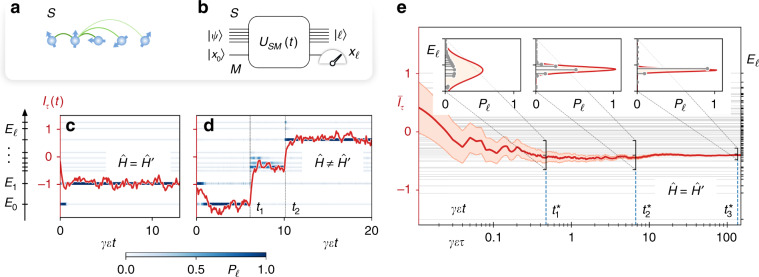
QND measurement of a many-body Hamiltonian $\hat{H}$ in a quantum simulator setting. The many-body spin system ${\mathcal{S}}$, shown in **a**, is entangled with an ancillary system ${\mathcal{M}}$ (meter) by the unitary ${\hat{U}}_{{\mathcal{SM}}}(t)=\exp \{-i{\int }_{0}^{t}{\mathrm{d}}{t}^{\prime}[\hat{H}\otimes {\mathbb{I}}+\vartheta ({t}^{\prime})\hat{H}\otimes \hat{P}]\}$. **b** Subsequent reading of a meter value ${x}_{\ell }\equiv {x}_{0}+{E}_{\ell }\int_{0}^{t}{\mathrm{d}}{t}^{\prime}\vartheta ({t}^{\prime})$ prepares the many-body system in an energy eigenstate $\left|\ell \right\rangle$ with the eigenvalue *E*_*ℓ*_. **c** Single trajectory simulation (4) and (5) of an ideal QND measurement for the Ising Hamiltonian (3) for *N* = 5 spins, *α* = 1.5, *h*∕*J* = 1.5. The window-filtered homodyne current ${{\mathcal{I}}}_{\tau }(t)$ (red curve) fluctuates around a value corresponding to the eigenenergy prepared by the measurement of $\hat{H}$. The thin horizontal lines show the system eigenenergies *E*_*ℓ*_ and the blue color indicates the conditional populations *P*_*ℓ*_(*t*) of the corresponding eigenstates. **d** Observation of quantum jumps due to the mismatch of the transverse fields ${\hat{H}}^{\prime}=\hat{H}+\delta \tilde{h}{\sum }_{j}{\hat{\sigma }}_{j}^{z}$ with $\delta \tilde{h}/J=-0.2$. The filtered photocurrent (red) clearly shows sudden jumps between eigenstates at times *t*_1_ and *t*_2_. **e** Preparation of energy eigenstates or microcanonical ensembles by the ideal QND measurement for *N* = 8 spins, *α* = 1.5, *h/J* = 0.8. The estimate of the system energy given by the cumulative time average of the homodyne current ${\overline{{\mathcal{I}}}}_{\tau }$ (red line) gradually converges to a single eigenenergy (gray lines) as averaging time *τ* increases. The corresponding uncertainty (red area) due to shot noise decreases as $\sim\! 1/\sqrt{\gamma \epsilon \tau }$. Inset: conditional population of the energy eigenstates (gray points) at times ${t}_{1}^{* }$, ${t}_{2}^{* }$, and ${t}_{3}^{* }$ is well captured by gaussian distributions of widths $J/\sqrt{4\gamma \epsilon {t}_{1,2,3}^{* }}$ describing the fluctuations of the shot noise averaged over $\tau ={t}_{1,2,3}^{* }$ (red curve). The SME is solved using the open-source QuTiP package^54^.

On a more formal level, we define QND measurement of a many-body Hamiltonian $\hat{H}$ as an indirect measurement by coupling the system of interest ${\mathcal{S}}$, illustrated in Fig. 1a, to an ancillary system ${\mathcal{M}}$ as meter. In a first step, the system is entangled with the meter according to the time evolution $U(t)=\exp (-i{\hat{H}}_{{\rm{QND}}}t)$ generated by the QND Hamiltonian

1 $${\hat{H}}_{{\rm{QND}}}=\vartheta \hat{H}\otimes \hat{P},$$

with coupling strength *ϑ* (*ℏ* = 1). To be specific and in light of examples below, we consider here as meter a continuous variable system with a pair of conjugated quadratures $\hat{X}$ and $\hat{P}$ obeying the canonical commutation relation $[\hat{X},\hat{P}]=i$. Consider now an initial state of the joint system prepared as $\left|\Psi \right\rangle =\left|\psi \right\rangle \otimes \left|{x}_{0}\right\rangle$, where $\left|\psi \right\rangle \equiv {\sum }_{\ell }{c}_{\ell }\left|\ell \right\rangle$ is a superposition of energy eigenstates, $\hat{H}\left|\ell \right\rangle ={E}_{\ell }\left|\ell \right\rangle ,$ and $\left|{x}_{0}\right\rangle$ is an (improper) eigenstate of $\hat{X}$ (or squeezed state). We obtain for the time-evolved state $\left|\Psi (t)\right\rangle =\hat{U}(t)\left|\psi \right\rangle \otimes \left|{x}_{0}\right\rangle ={\sum }_{\ell }{c}_{\ell }\left|\ell \right\rangle \otimes \left|{x}_{0}+\vartheta {E}_{\ell }t\right\rangle$. Reading the meter as *x*_*ℓ*_ ≡ *x*_0_ + *ϑE*_*ℓ*_*t*, and thus measuring the eigenvalue *E*_*ℓ*_, will prepare the system in $\left|\ell \right\rangle$ (or in the relevant subspace in case of degeneracies). The probability for obtaining the particular measurement outcome *E*_*ℓ*_ is *P*_*ℓ*_ =  ∣*c*_*ℓ*_∣^2^. Repeating the QND measurement will reproduce the particular *E*_*ℓ*_ with certainty, with the system remaining in $\left|\ell \right\rangle$. The above discussion is readily extended to mixed initial system states, and to initial meter states, for example, as coherent states.

In an analog quantum simulator setting, QND measurement of the many-body Hamiltonian $\hat{H}$ is incorporated by engineering the extended system-meter Hamiltonian ${\hat{H}}_{{\mathcal{SM}}}=\hat{H}\otimes {\mathbb{I}}+\vartheta \hat{H}\otimes \hat{P}$. In an interaction picture with respect to $\hat{H}\otimes {\mathbb{I}}$, the joint system then evolves according to the Hamiltonian ${\hat{H}}_{{\mathcal{SM}}}^{{\rm{int}}}\equiv {\hat{H}}_{{\rm{QND}}}$ realizing the QND measurement discussed above and illustrated in Fig. 1b. On the other hand, by allowing the system-meter coupling *ϑ*(*t*) to be switched on and off in time, we can alternate between the conventional free-evolution simulation and QND measurement mode of the system. In an actual implementation, as discussed below for trapped ions, we will achieve building the extended system-meter Hamiltonian

2 $${\hat{H}}_{{\mathcal{SM}}}=\hat{H}^{\prime} \otimes {\mathbb{I}}+\vartheta \hat{H}\otimes \hat{P},$$

where $\hat{H}^{\prime}$ and $\hat{H}$ may differ (slightly). We note that the QND measurement of $\hat{H}$ is obtained by fine-tuning $\hat{H}^{\prime}=\hat{H}$. A mismatch $\hat{H}^{\prime} \,\ne\, \hat{H}$ will be visible as quantum jumps between energy eigenstates in repeated measurements.

In the trapped-ion example discussed below the many-body Hamiltonian $\hat{H}$ will be a long-range transverse Ising model^30–33^,

3 $$\hat{H}=-\sum _{i < j}^{N}{J}_{ij}{\hat{\sigma }}_{i}^{x}{\hat{\sigma }}_{j}^{x}-h\mathop {\sum}\limits _{j}^{N}{\hat{\sigma }}_{j}^{z},$$

where *J*_*ij*_  = *J* ∕ ∣*i* − *j*∣^*α*^ with 0 < *α* < 3 and *h* the transverse field. Remarkably, in our implementation, the Hamiltonian $\hat{H}^{\prime}$ will differ from $\hat{H}$ just by the transverse field taking on the value $h^{\prime}$. We will be able to tune $h=h^{\prime}$, thus achieving the QND condition.

As the last step in our formal development, we wish to formulate QND measurement of $\hat{H}$ as measurement continuous in time^34–37^. Physically, this amounts to making frequent and, in a continuum limit, continuous readouts *X*(*t*) of the meter variable $\hat{X}$, with the quantum many-body system evolving according to (2). Following a well-established formalism of quantum optics^38,39^, we write for the system under continuous observation a stochastic master equation (SME) for a conditional density matrix ${\hat{\rho }}_{{\rm{c}}}(t)$ of the many-body system. In our context this SME reads

4 $${\mathrm{d}}{\hat{\rho }}_{{\rm{c}}}(t)=	 -i[\hat{H}^{\prime} ,{\hat{\rho }}_{{\rm{c}}}(t)]{\mathrm{d}}t+\gamma {\mathcal{D}}[\hat{H}/J]{\hat{\rho }}_{{\rm{c}}}(t)\ {\mathrm{d}}t\\ 	+\sqrt{\gamma \epsilon }{\mathcal{H}}[\hat{H}/J]{\hat{\rho }}_{{\rm{c}}}(t)\ {\mathrm{d}}W(t),$$

5 $${\mathrm{d}}X(t)\equiv I(t){\mathrm{d}}t=2\sqrt{\gamma \epsilon }{\langle \hat{H}/J\rangle }_{{\rm{c}}}{\mathrm{d}}t+{\mathrm{d}}W(t),$$

with d*W*(*t*) a Wiener increment, to be interpreted as an Itô stochastic differential equation. In a quantum optical setting, as in the ion trap example below, *I*(*t*) is identified with photocurrent in homodyne detection of scattered light^38^. Monitoring the photocurrent $I(t) \sim {\langle \hat{H}\rangle }_{{\rm{c}}}$ thus provides continuous read-out of the many-body Hamiltonian $\hat{H}$ with ${\left\langle \ldots \right\rangle }_{{\rm{c}}}\equiv {\rm{Tr}}[\ldots {\hat{\rho }}_{{\rm{c}}}(t)]$ up to shot noise. Thus, ${\hat{\rho }}_{{\rm{c}}}(t)$ describes the many-body quantum state conditional to observing a particular photocurrent trajectory *I*(*t*), as can be observed in a single run of an experiment. In (4) and (5) *γ* is an effective measurement rate, and *ϵ* is a measurement efficiency. Furthermore, we have defined a Lindblad superoperator ${\mathcal{D}}[\hat{s}]{\hat{\rho }}_{{\rm{c}}}\equiv \hat{s}{\hat{\rho }}_{{\rm{c}}}{\hat{s}}^{\dagger }-({\hat{s}}^{\dagger }\hat{s}{\hat{\rho }}_{{\rm{c}}}+{\rm{H.c.}})/2$ describing decoherence due to the quantum measurement backaction, and the nonlinear superoperator ${\mathcal{H}}[\hat{s}]{\hat{\rho }}_{{\rm{c}}}\equiv (\hat{s}-{\langle \hat{s}\rangle }_{{\rm{c}}}){\hat{\rho }}_{{\rm{c}}}+{\rm{H.c.}}$, which updates the density matrix conditioned on the observation of the homodyne photocurrent. Finally, not reading the meter, that is, averaging overall measurement outcomes *I*(*t*), the SME (4) reduces to a master equation with Lindblad term $\sim\! {\mathcal{D}}[\hat{H}]\hat{\rho }$, that is, realizing a reservoir coupling with jump operator $\hat{H}$, which erases all off-diagonal terms of the averaged density matrix $\hat{\rho }$ in the energy eigenbasis.

Equations (4) and (5) allow us to simulate single measurement runs corresponding to a stochastic trajectory *I*(*t*). Figure 1c illustrates ideal QND measurement, $\hat{H}^{\prime} =\hat{H}$, of the Hamiltonian (3) by plotting a sample trajectory of a filtered photocurrent, obtained by averaging *I*(*t*) over a time window *τ*, ${{\mathcal{I}}}_{\tau }(t)={(2N\sqrt{\gamma \epsilon }\tau )}^{-1}{\int }_{0}^{\infty }{\mathrm{d}}t^{\prime} I(t-t^{\prime} ){e}^{-t^{\prime} /\tau }$. As initial condition we take all spins pointing against the transverse field. As seen in Fig. 1c the trajectory ${{\mathcal{I}}}_{\tau }(t)$ (red curve) stabilizes on a time scale  ~*γ*^−1^ on a particular energy eigenvalue *E*_*ℓ*_ of (3) (up to fluctuations from shot noise). In this figure we consider and show only the eigenstates and eigenenergies (thin horizontal lines) within the symmetry sector containing the ground state of the Ising model with *J*, *h* >  0, see Methods. The collapse, and thus preparation of the many-body wavefunction in the corresponding energy eigenstate, is indicated by plotting the populations ${P}_{\ell }(t)\equiv \left\langle \ell \right|{\hat{\rho }}_{{\rm{c}}}(t)\left|\ell \right\rangle$ (blue shadings in Fig. 1c). Figure 1d shows quantum jumps between energy eigenstates induced by $\hat{H}^{\prime}\, \ne\, \hat{H}$. For weak perturbation ($\left|\right.[\hat{H}^{\prime} ,\hat{H}]\left|\right.\ll | \hat{H}{| }^{2}$) there are rare jumps between the energy eigenstates, indicated as *t*_1_ and *t*_2_ for the trajectory in Fig. 1d. Finally, Fig. 1e plots the integrated current ${\overline{{\mathcal{I}}}}_{\tau }={(2N\sqrt{\gamma \epsilon }\tau )}^{-1}{\int }_{0}^{\tau }I(t){\mathrm{d}}t$ and its fluctuations as a function of total integration time *τ*. For *N* = 8 spins starting in a thermal state, the integrated current ${\overline{{\mathcal{I}}}}_{\tau }$ (red curve) exhibits a collapse at a rate  ~*γ* to a particular energy eigenstate. The insets shows the probabilities *P*_*ℓ*_ for various times, and the narrowing of the energy resolution as $\Delta E/J \sim 1/\sqrt{\gamma \epsilon \tau }$ with growing *τ* (see Methods); first to small energy window containing a few eigenstates as in a microcanonical ensemble, and eventually to a single energy eigenstate.

### Implementation with trapped ions

**Fig. 2:**
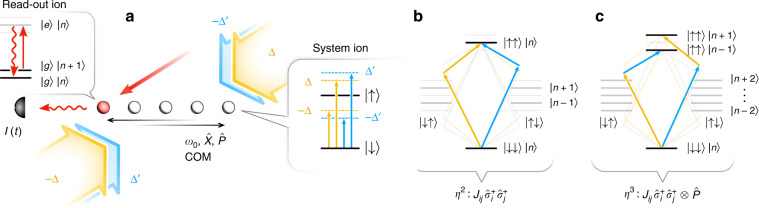
Trapped-ion implementation of the system-meter Hamiltonian ${\hat{H}}_{{\mathcal{SM}}}$. **a** Ion string with *N* system ions (white) illuminated by four laser beams in a double Mølmer–Sørensen configuration. As described in the text this generates ${\hat{H}}_{{\mathcal{SM}}}$ [see Eq. (2)] with transverse Ising Hamiltonians $\hat{{H}^{\prime}}$ (6) and $\hat{H}$ (3), and the meter variable $\hat{P}$ representing the COM motion. The meter variable $\hat{X}$ is read by driving one, or potentially several ancilla ions (red) with a laser (red beam) tuned to the red motional COM sideband (see text). Homodyne detection of the scattered light to read $\hat{X}$, and thus revealing $\hat{H}$ in the photocurrent $I(t) \sim {\langle \hat{H}\rangle }_{{\rm{c}}}$ [see Eq. (5)]. **b** Level scheme of a pair of ions sharing the COM phonon mode, illustrating one of the elementary processes contributing to the Ising term $-{\sum }_{i < j}{J}_{ij}{\hat{\sigma }}_{i}^{x}{\hat{\sigma }}_{j}^{x}\otimes {\mathbb{I}}$ in second order in *η*. **c** Level scheme showing the corresponding third-order processes contributing to $-\vartheta {\sum }_{i < j}{J}_{ij}{\hat{\sigma }}_{i}^{x}{\hat{\sigma }}_{j}^{x}\otimes \hat{P}$ (see text).

We now provide a trapped-ion implementation of the system-meter Hamiltonian ${\hat{H}}_{{\mathcal{SM}}}$ (2). As shown in Fig. 2a, we consider a string of *N* ions in a linear Paul trap representing spin-1/2 $\left\{{\left|\downarrow \right\rangle }_{i},{\left|\uparrow \right\rangle }_{i}\right\}$. These two-level ions can be driven by laser light $\left|\downarrow \right\rangle \to \left|\uparrow \right\rangle$, where the recoil associated with absorption and emission of photons provides a coupling to vibrational eigenmodes of the ion chain. This includes in particular the center-of-mass motion (COM) with $\hat{X}$ and $\hat{P}$ position and momentum operators, respectively, which play the role of meter variables.

To generate in ${\hat{H}}_{{\mathcal{SM}}}$ both the Ising interaction $-{\sum }_{i < j}\, {J}_{ij}{\hat{\sigma }}_{i}^{x}{\hat{\sigma }}_{j}^{x}\otimes {\mathbb{I}}$, as well as the Ising term coupled to COM, $-\vartheta {\sum }_{i < j}\, {J}_{ij}{\hat{\sigma }}_{i}^{x}{\hat{\sigma }}_{j}^{x}\otimes \hat{P}$, we choose a laser configuration consisting of two pairs of counterpropagating laser beams (cf. Fig. 2a). In generalization of refs. ^40,41^, we call this a double Mølmer–Sørensen configuration. The first pair of MS beams (shown as amber in Fig. 2) is detuned by  ±Δ from ionic resonance, while the second pair (blue) is detuned by $\pm \!\Delta ^{\prime}$. Furthermore, we choose $\Delta ^{\prime} -\Delta ={\omega }_{0}$ with *ω*_0_ the COM frequency. These four laser beams give rise to laser-induced two-photon processes involving pairs of ions, which are depicted in Fig. 2b, c).

First, as shown in Fig. 2b, absorption of a photon from the one of the amber MS laser beam followed by an absorption from the counterpropagating amber beam gives rise to a two-photon excitation $\left|\downarrow \downarrow \right\rangle \to \left|\uparrow \uparrow \right\rangle$, which is resonant with twice the (bare) ionic transition frequency of the two-level ion. We emphasize that this process leaves the motional state of the ion chain unchanged, as illustrated by $\left|n\right\rangle \to \left|n\right\rangle$ for the COM mode, with *n* the phonon occupation number. This process will thus contribute a term $\sim {\hat{\sigma }}_{i}^{+}{\hat{\sigma }}_{j}^{+}$ to the effective spin–spin interaction. The second pair of MS beams (blue) will again contribute a resonant two-photon excitation, which adds coherently to the first contribution. By considering all possible processes, we obtain the effective Ising interaction $-{\sum }_{i < j}{J}_{ij}{\hat{\sigma }}_{i}^{x}{\hat{\sigma }}_{j}^{x}\otimes {\mathbb{I}}$ in ${\hat{H}}_{{\mathcal{SM}}}$. An explicit expression for *J*_*ij*_ is given in Methods in second-order perturbation theory in the Lamb–Dicke parameter $\eta =k/\sqrt{2m{\omega }_{0}}\ll 1$, where *m* is the ion mass and *k* is the magnitude of the laser wavevector.

Second, with the choice $\Delta ^{\prime} -\Delta ={\omega }_{0}$ two-photon processes involving absorption from an amber MS beam and a blue MS beam will be detuned by the COM frequency from two-photon resonance, that is, be resonant with the motional sidebands  ±*ω*_0_ (cf. Fig. 2c). These processes will change the phonon number by one, and by considering all possible processes contribute a term $-\vartheta {\sum }_{i < j}{J}_{ij}{\hat{\sigma }}_{i}^{x}{\hat{\sigma }}_{j}^{x}\otimes \hat{P}$ to ${\hat{H}}_{{\mathcal{SM}}}$. Here $\vartheta \simeq -\eta \sqrt{2/N}$, and *J*_*ij*_ is identical to the couplings obtained above. We note that this term is of order *η*^3^ (for details see Methods).

By considering a (small) imbalance of Rabi frequencies in MS laser configurations, we can create in ${\hat{H}}_{{\mathcal{SM}}}$ a transverse-field term $-h{\sum }_{j}{\hat{\sigma }}_{j}^{z}\otimes {\mathbb{I}}$, and in addition a term $+\vartheta h{\sum }_{j}{\hat{\sigma }}_{j}^{z}\otimes \hat{P}$ (see Methods and Supplementary Note I). Thus, our laser configuration generates $\hat{H}$ and $\hat{H}^{\prime}$ with the same Ising term, but opposite transverse field  ±*h*. To rectify the transverse-field mismatch, we can offset the detuning of the four lasers by a small amount $\pm\! {\Delta }^{({\prime} )}\to \pm\! {\Delta }^{({\prime} )}-2B$. We obtain $\hat{H}$ as in Eq. (3) and

6 $$\hat{H}^{\prime} =-\mathop {\sum}\limits _{i < j}^{N}{J}_{ij}{\hat{\sigma }}_{i}^{x}{\hat{\sigma }}_{j}^{x}-(B-h)\mathop {\sum}\limits _{j}^{N}{\hat{\sigma }}_{j}^{z}.$$

The choice *B* =  2*h* thus allows us to tune to the QND sweetspot $\hat{H}^{\prime} =\hat{H}$ as in Fig. 1c, e, while away from this point we obtain $\hat{H}^{\prime}\, \ne\, \hat{H}$ as considered in Fig. 1d.

Finally, the homodyne current (5) corresponding to a continuous measurement of the COM quadrature $\hat{X}$, and thus of the Hamiltonian $\hat{H}$, can be measured via homodyne detection of the scattered light from an ancillary ion driven by a laser on the red motional COM sideband (cf. Fig. 2a and Methods).

### QND measurement protocols

Implementation of ${\hat{H}}_{{\mathcal{SM}}}$ with time-dependent system-meter coupling *ϑ*(*t*) allows protocols where we switch between time windows of unobserved quantum simulation, and measurement of energy, and thus preparation of energy eigenstates, which is verified by observing convergence of the filtered photocurrent. In addition, the Hamiltonians (3) and (6) can be made time dependent, for example, with a time-dependent magnetic field. This allows us to perform work on the system, and measure work distribution functions via measurement of energy^25^. Our QND toolbox thus opens up the door to address experimentally fundamental problems of (non-equilibrium) statistical mechanics in analog quantum simulation. We apply the QND toolbox below first to ETH^42,43^ and then we consider testing QFRs^25^ in interacting many-body systems. We emphasize that our setting explores naturally the interesting regime of mesoscopic particle numbers from a few to tens of spins.

### Thermal properties of energy eigenstates

Single energy eigenstates $\left|\ell \right\rangle$ can encode thermal properties, which we typically associate with a microcanonical or canonical ensemble describing systems in thermodynamic equilibrium. This eigenstate thermalization concerns, on the one hand, expectation values of few-body observables, leading to the remarkable prediction of the ETH that diagonal matrix elements $\left\langle \ell | \hat{O}| \ell \right\rangle$ have to agree with the microcanonical average at energy *E*_*ℓ*_, $\left\langle \ell | \hat{O}| \ell \right\rangle =O({E}_{\ell })={\rm{tr}}(\hat{O}{\hat{\rho }}_{{E}_{\ell }}^{{\rm{mc}}})$. Here ${\hat{\rho }}_{{E}_{\ell }}^{{\rm{mc}}}$ is the microcanonical density operator as a mixture of energy eigenstates within a narrow range centered around *E*_*ℓ*_. On the other hand, ETH imposes constraints on dynamical properties for diagonal and off-diagonal matrix elements $\left\langle \ell ^{\prime} | \hat{O}| \ell \right\rangle$; for example, two-time correlation functions and dynamical susceptibilities have to be related by the fluctuation-dissipation theorem^42^. To be more specific, ETH suggests a structure^44^ $\left\langle \ell ^{\prime} | \hat{O}| \ell \right\rangle =O(\overline{E}){\delta }_{\ell ^{\prime} \ell }+{e}^{-S(\overline{E})/2}{f}_{\hat{O}}(\overline{E},\omega ){R}_{\ell ^{\prime} \ell }$, where diagonal and off-diagonal matrix elements are determined by the functions $O(\overline{E})$ and ${f}_{\hat{O}}(\overline{E},\omega )$, respectively, which depend smoothly on their arguments $\overline{E}=({E}_{\ell }+{E}_{\ell ^{\prime} })/2$ and $\omega ={E}_{\ell ^{\prime} }-{E}_{\ell }$. $S(\overline{E})$ is the thermodynamic entropy at the mean energy $\overline{E}$, and ${R}_{\ell ^{\prime} \ell }$ is a random number with zero mean and unit variance. An experimental test of ETH, therefore, requires the ability to measure both diagonal and off-diagonal elements, something that is provided by our ion toolbox.

The transverse Ising model (3), as realized with ions, provides a rich testbed for ETH^45^. For 1 < *α* ≤ 2, this model features a ferromagnetic transition at finite temperature or energy density, in a canonical or microcanonical description, respectively. As illustrated above in Fig. 1, our trapped-ion QND toolbox enables the preparation of microcanonical ensembles of variable width Δ*E*. According to ETH, the ferromagnetic transition persists even in the limit of vanishing Δ*E*, which corresponds to the preparation of a single energy eigenstate.

**Fig. 3:**
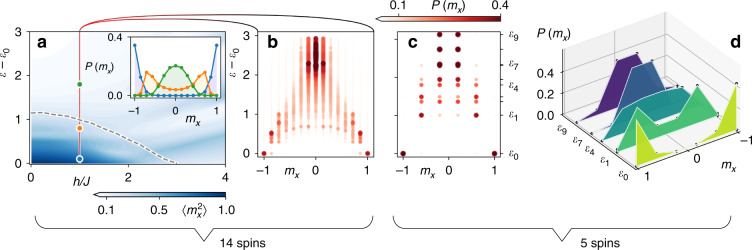
Excited-state phase transition in the Ising model (3) with *α* = 1.5. **a** Ferro-paramagnet crossover in the Ising model of *N* = 14 spins prepared by the energy measurements in microcanonical ensembles of width Δ*E*∕(*JN*) = 0.1. The transition between magnetically ordered phase ${\langle {\hat{m}}_{x}^{2}\rangle }_{\text{mc}}\approx 1$ (dark blue) to disordered phase ${\langle {\hat{m}}_{x}^{2}\rangle }_{\text{mc}}\approx 0$ (light blue) is shown as function of the mean energy density $\varepsilon ={\langle \hat{H}\rangle }_{{\rm{mc}}}/(JN)$ and the transverse field *h*. An estimate of the critical energy density in the thermodynamic limit, obtained with Monte-Carlo simulation (we use the ALPS code^55^) of canonical ensembles of 512 spins with rescaled interactions (see Methods and Supplementary Note II), is shown as black dashed line. The inset shows the order parameter distribution $P\left({m}_{x}\right)$ for *h*∕*J* = 1 and *ε* = 0.1, 0.8, and 1.8 in blue, orange, and green, respectively. Test of ETH (within the symmetry sector $\left\{+1,-1\right\}$ see Methods): **b** order–disorder transition is seen as crossover from bi-modal distribution of $P\left({m}_{x}\right)$ at low energies to a single-peak distribution at high energies, shown on the level individual eigenstates. Color intensity and the dot size indicate the corresponding probability. **c**, **d** Qualitatively similar energy dependence of $P\left({m}_{x}\right)$ shown for a system of just five spins. **d** Signatures of the phase transition visible for representative sample of eigenstates.

For reference, the microcanonical phase diagram at finite Δ*E* is shown in Fig. 3a for an experimentally accessible system size of *N* = 14 spins and *α* = 1.5 (see Supplementary Note I for experimental parameters, we use QuSpin for the exact diagonalization^46^). The ferromagnetic transition is clearly manifest in the distribution *P*(*m*_*x*_) of the magnetization ${\hat{m}}_{x}={N}^{-1}{\sum }_{j}{\hat{\sigma }}_{j}^{x}$, which is bi-modal in the ferromagnetic phase, see the inset in Fig. 3a. Consequently, fluctuations $\left\langle {\hat{m}}_{x}^{2}\right\rangle$ are finite (vanish) in the ferromagnetic (paramagnetic) phase, and indicate order even in the absence of symmetry-breaking fields. A trapped-ion quantum simulator provides the ability to perform single-site resolved read-out of spins, thus giving direct access to the distribution *P*(*m*_*x*_) and, consequently, the fluctuations $\left\langle {\hat{m}}_{x}^{2}\right\rangle$. Due to the quasi-diagonal structure of $\left\langle \ell ^{\prime} | \hat{O}| \ell \right\rangle$ for ETH-satisfying observables $\hat{O}$, the hypothesis is expected to hold for any power of such observables and, in particular, also for the full probability distribution function *P*(*m*_*x*_)^44^. Indeed, as we show in Fig. 3b–d, respectively, we find clear signatures of the transition in *P*(*m*_*x*_) for individual energy eigenstates both for *N*  = 14 and even much smaller system of only *N* =  5 spins, in which single eigenenergies can be resolved with current experimental technology (see Supplementary Note I).

The observation of the Ising transition in single eigenstates gives a qualitative indication of eigenstate thermalization in diagonal matrix elements. A stringent quantitative assessment requires to show that fluctuations of single-eigenstate expectation values $\left\langle \ell | \hat{O}| \ell \right\rangle$ around the microcanonical average ${\rm{tr}}(\hat{O}{\hat{\rho }}_{{E}_{\ell }}^{{\rm{mc}}})$ are suppressed with increasing system size^42^. We discuss experimental requirements for such a test, along with a protocol to measure off-diagonal matrix elements, in Supplementary Note III.

### Work distribution function and QFRs

Projective measurements in the energy eigenbasis are the key ingredient for the long-sought experimental verification of QFRs^25^. The challenging requirement to measure changes in the energy of the system on the level of single energy eigenstates has been achieved only recently in single-particle systems^47,48^. Our QND measurement scheme opens up the possibility to probe QFRs in a true many-body setting.

As an illustration we consider the celebrated Jarzynski equality, which describes the mean value of the exponentiated work performed on a system in an arbitrary non-equilibrium process defined by a time-dependent Hamiltonian $\hat{H}(t)$^25^. The equality relates the work to the difference between the free energies Δ*F* of equilibrium systems described by the Hamiltonian at the initial *t*_0_ and the final *t*_1_ times:

7 $$\left\langle {e}^{-\beta W}\right\rangle ={e}^{-\beta \Delta F}.$$

Here *β* is the inverse temperature specifying an initial canonical thermal state of the system ${\hat{\rho }}_{\beta }={e}^{-\beta \hat{H}({t}_{0})}/{Z}_{{t}_{0}}$ with ${Z}_{{t}_{0}}={\rm{tr}}[{e}^{-\beta \hat{H}({t}_{0})}]$ and $\Delta F={F}_{{t}_{1}}-{F}_{{t}_{0}}=-{\mathrm{ln}}({Z}_{{t}_{1}}/{Z}_{{t}_{0}})/\beta$. The average on the left-hand side of Eq. (7) is performed with respect to the distribution of work ${\mathcal{P}}(W)$ (see Methods). The work itself is determined as the difference between the outcomes of two energy measurements before and after the time-dependent protocol $W\equiv {E}_{\ell ^{\prime} }^{{t}_{1}}-{E}_{\ell }^{{t}_{0}}$. Remarkably, while the work distribution does depend on details of the time evolution given by $\hat{H}(t)$, the average is defined only by the initial and final Hamiltonians. Therefore, the QFR enables experimental measurements of the equilibrium property Δ*F* via measurement of work in a non-equilibrium process.

Our scheme provides the required ingredients for probing the QFR in the interesting regime of intermediate system sizes, which is dominated by quantum fluctuations: as presented above, the scheme allows for independent temporal control of parameters of the spin Hamiltonian as well as the system-meter coupling in Eq. (2). Further, single energy levels are well resolved for a system of five interacting spins as we consider in the following (see Supplementary Note I for experimental parameters).

**Fig. 4:**
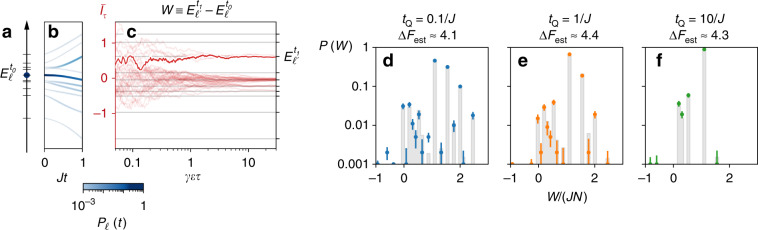
Verification of Jarzynski equality (7) for the transverse-field Ising model with five spins. A single realization of the proposed protocol consists of **a** the projection of the initial thermal state of spins to an eigenstate ${E}_{\ell }^{{t}_{0}}$ of the initial Hamiltonian with transverse-field value ${h}_{{t}_{0}}$, **b** the free evolution under a linear change of the transverse field *h* during time *t*_Q_ = *t*_1_ − *t*_0_ (probabilities *P*_*ℓ*_(*t*) to populate the instantaneous energy eigenstates are shown in shades of blue), **c** The final energy read-out ${E}_{\ell }^{{t}_{1}}$ of the system at transverse-field ${h}_{{t}_{1}}$ (photocurrent realizations for simulated experimental runs are shown in red), resulting in the work *W* (see text) performed during the non-equilibrium process. **d**–**f** The resulting work probability distribution ${\mathcal{P}}(W)$ for ${h}_{{t}_{0}}=2J$, ${h}_{{t}_{1}}=0.5J$, *α* = 2, *β* = 0.5∕*J*, and various quench times *t*_Q_. The gray bars show theoretical probability as a function of work *W*. The color dots with the corresponding vertical error bars (one standard deviation) show the estimated probabilities for 1000 simulated experimental runs. Independent of the quench duration the Jarzynski relation (7) yields similar estimations of the free energy difference Δ*F*_est_ (fluctuating due to the finite number of runs) with the true value given by Δ*F* ≈ 4.35.

This enables the protocol shown in Fig. 4a–c, in which a first measurement of the energy is carried out while the magnetic field *h* in Eq. (3) is kept constant at a value ${h}_{{t}_{0}}$. Then, the system-meter coupling is switched off, and the magnetic field is linearly ramped to a final value ${h}_{{t}_{1}}$, followed by another measurement. The statistics of corresponding measurement outcomes, ${E}_{\ell }^{{t}_{0}}$ and ${E}_{\ell ^{\prime} }^{{t}_{1}}$, determines the distribution of work ${\mathcal{P}}(W)$ performed on the system during the magnetic field ramp. Initialization of the system at arbitrary temperatures can be emulated by weighting different runs of the protocol according to the Gibbs distribution ${e}^{-\beta {E}_{\ell }^{{t}_{0}}}/{Z}_{{t}_{0}}$ with the initial energy ${E}_{\ell }^{{t}_{0}}$.

The resulting work distributions ${\mathcal{P}}(W)$ for various quench durations *t*_Q_ are shown in Fig. 4d–f. While the probability distribution for fast (blue dots) and slow (green dots) quenches differs significantly the estimated free energy difference $\Delta {F}_{{\rm{est}}}=-{\mathrm{ln}}\langle {e}^{-\beta W}\rangle /\beta$ approximately (due to the finite number of simulated experimental runs) matches the true value of Δ*F* for all three quench speeds; thus, verifying the equality (7).

## Discussion

We have developed a QND toolbox in analog quantum simulation realizing single-shot measurement of the energy of an isolated quantum many-body system, as a key element towards experimental studies in non-equilibrium quantum statistical mechanics. This comprises ETH and quantum thermodynamics, including quantum work distribution, and Jarzynski and Crooks fluctuations relations^25^ in mesoscopic quantum many-body systems. The present work outlines an ion-trap implementation with COM phonons as meter. However, the concepts and techniques carry over to other platforms including CQED with atoms^49^ and superconducting qubits^50^, where the role of the meter can be represented by cavity photons read with homodyne detection, and Rydberg tweezer arrays^8–12^ by coupling to a small atomic ensemble encoding the continuous meter variables^51^, respectively. Finally, while the present work considers QND measurement of the total Hamiltonian $\hat{H}$ of an isolated system, our approach generalizes to measuring Hamiltonians ${\hat{H}}_{\mathrm{A}}$ of subsystems, as is of interested in quantum transport of energy, or energy exchange in coupling the many-body system of interest to a bath.

## Methods

### System-meter coupling Hamiltonian

We choose for the four lasers in our double MS configuration the detuning and the Rabi frequency as (Δ, Ω), (− Δ, Ω + δΩ), $\left({\Delta }^{\prime},\Omega \right)$, $\left(-{\Delta }^{\prime},\Omega +\delta \Omega \right)$, where *δ*Ω ∝ *η*^2^Ω is a small imbalance we use to generate the transverse-field term in the spin model. We are interested in the regime of sufficiently large detunings compared to the Rabi frequency Ω, such that single lasers only virtually excite the ions and the phonon modes, $\Omega \ll {\Delta }^{({\prime} )}$, ${\eta }_{q}\Omega \ll | {\Delta }^{({\prime} )}-{\omega }_{q}|$, where *ω*_*q*_ is the oscillation frequency of the *q*th phonon mode and ${\eta }_{q}\equiv \eta \sqrt{{\omega }_{0}/{\omega }_{q}}$. On large timescales $t\gg 1/{\Delta }^{({\prime} )},1/{\omega }_{0}$, we obtain an effective Hamiltonian ${\hat{H}}_{{\mathcal{SM}}}$ describing the coupled dynamics of the system and the meter, that is, the spins and the COM phonon mode, by performing the Magnus expansion^52^ to the time evolution operator in the interaction picture (see Supplementary Note I). We further expand ${\hat{H}}_{{\mathcal{SM}}}$ in terms of *η*. In second order in *η* we recover the transverse-field Ising Hamiltonian^30–32^, ${\hat{H}}_{{\mathcal{SM}}}^{\left(2\right)}=(-\sum _{i < j}{J}_{ij}{\hat{\sigma }}_{i}^{x}{\hat{\sigma }}_{j}^{x}+h\sum _{j}{\hat{\sigma }}_{j}^{z})\otimes {\mathbb{I}}\equiv \hat{H}^{\prime} \otimes {\mathbb{I}}$, where the spin–spin couplings

8 $${J}_{ij}=-{\eta }^{2}{\omega }_{0}\mathop {\sum}\limits_{q}{M}_{iq}{M}_{jq}\left[\frac{{\Omega }^{2}}{{\Delta }^{2}-{\omega }_{q}^{2}}+\frac{{\Omega }^{2}}{{({\Delta }^{\prime})}^{2}-{\omega }_{q}^{2}}\right]$$

include contributions from the two MS configurations independently with *M*_*iq*_ denoting the distribution matrix element of the *q*th phonon mode. The transverse-field strength is $h=\Omega \delta \Omega \left(1/\Delta +1/{\Delta }^{\prime}\right)/2$.

Crucially, under the condition ${\Delta }^{\prime}-\Delta ={\omega }_{0}$, the crosstalk between the two MS configurations leads to an extra resonant processes as exemplified by Fig. 2c. These are described by expanding the effective Hamiltonian ${\hat{H}}_{{\mathcal{SM}}}$ to third order in *η*, ${\hat{H}}_{{\mathcal{SM}}}^{\left(3\right)}=(-\eta \sqrt{2}{M}_{i0})(-{\sum }_{i < j}{J}_{ij}{\hat{\sigma }}_{i}^{x}{\hat{\sigma }}_{j}^{x}-h{\sum }_{j}{\hat{\sigma }}_{j}^{z})\otimes \hat{P}\equiv \vartheta \hat{H}\otimes \hat{P}$, where ${M}_{i0}\simeq 1/\sqrt{N}$ is the (equal) distribution matrix element of the COM mode. Combining ${\hat{H}}_{{\mathcal{SM}}}^{\left(2\right)}$ and ${\hat{H}}_{{\mathcal{SM}}}^{\left(3\right)}$ gives the desired system-meter Hamiltonian (2). The transverse field in ${\hat{H}}_{{\mathcal{SM}}}^{\left(2\right)}$ and ${\hat{H}}_{{\mathcal{SM}}}^{\left(3\right)}$ can be independently tuned with the method discussed in the main text. Higher order terms beyond ${\hat{H}}_{{\mathcal{SM}}}^{\left(3\right)}$ have negligible effects, for details see Supplementary Note I.

Our double MS configuration can be implemented with both axial and transverse phonon modes. The implementation with transverse modes gives rise to power-law spin interactions *J*_*ij*_ = *J* ∕ ∣*i* −  *j*∣^*α*^ with 0 ≤ *α* ≤ 3, which are considered in the rest of this paper. Experimental considerations and scalability are discussed in the Supplementary Note I.

### Continuous read-out of $\hat{X}$

We assume that in Fig. 2 the ancillary ion does not see the four MS lasers (amber and blue) and, similarly, the system ions do not couple to the read-out laser (red), that is, we assume single-ion addressability^53^ or with mixed species^5^. The read-out laser is tuned in resonance with the red sideband of the COM mode, Δ_e_ = *ω*_0_, under the resolved-sideband condition *ω*_0_ ≫ Γ_e_, Ω_0_, where Γ_e_ is the spontaneous emission rate of the cooling transition $\left|e\right\rangle \to \left|g\right\rangle$, while Ω_0_ and Δ_e_ are the Rabi frequency and the detuning of the cooling laser, respectively. In this regime, the emitted electric field is proportional to $\langle {\hat{a}}_{0}\rangle$ with ${\hat{a}}_{0}$ the annihilation operator of the COM mode. Homodyne detection then directly reveals the quadrature of the COM phonon (the meter). The homodyne current can be written as (see Supplementary Note I)

9 $${\mathrm{d}}X(t)\equiv I(t){\mathrm{d}}t=\sqrt{2\epsilon {\gamma }_{{\rm{s}}}}{\langle \hat{X}\rangle }_{{\rm{c}}}+{\mathrm{d}}W(t),$$

where *ϵ* is the photon detection efficiency, ${\gamma }_{{\rm{s}}}\simeq {k}_{0}^{2}{\Omega }_{0}^{2}/(2{\Gamma }_{{\rm{e}}}N{m}_{0}{\omega }_{0})$ is the measurement rate with *k*_0_ the cooling laser wavevector and *m*_0_ the ancillary ion mass, and we have chosen the frequency and phase of the homodyne local oscillator to maximize the homodyne current. Correspondingly, the evolution of the conditional state ${\rho }_{{\rm{c}}}^{{\mathcal{SM}}}(t)$ of spin system plus the meter is described by a SME

10 $${\mathrm{d}}{\hat{\rho }}_{{\rm{c}}}^{{\mathcal{SM}}}(t)=	 -i[{\hat{H}}_{{\mathcal{SM}}},{\hat{\rho }}_{{\rm{c}}}^{{\mathcal{SM}}}(t)]{\mathrm{d}}t+{\gamma }_{{\rm{s}}}{\mathcal{D}}\left[{\hat{a}}_{0}\right]{\hat{\rho }}_{{\rm{c}}}^{{\mathcal{SM}}}(t){\mathrm{d}}t\\ \, 	+\sqrt{\epsilon {\gamma }_{{\rm{s}}}}{\mathcal{H}}\left[{\hat{a}}_{0}\right]{\hat{\rho }}_{{\rm{c}}}^{{\mathcal{SM}}}(t){\mathrm{d}}W(t),$$

Eliminating the meter under the condition *γ*_s_  ≫  ∣*ϑJ*∣, we realize continuous QND read-out of the spin Hamiltonian as described by Eqs. (4) and (5) with *γ*  =  2(*ϑJ*)^2^∕*γ*_s_.

We further emphasize that the read-out laser, which is tuned to the red sideband, also acts as cooling of the COM mode. Furthermore, the read-out signal can be enhanced with several ancilla ions.

### Energy measurement resolution

Here we estimate the signal-to-noise ratio (SNR), which allows us to distinguish two adjacent energy levels separated by Δ*E*. The difference of photocurrents (5) corresponding to the two energy levels integrated over time *τ* reads

$$\int_{0}^{\tau}[{I}_{1}(t)-{I}_{2}(t)]{\mathrm{d}}t = \underbrace{2\sqrt{\gamma \epsilon }(\Delta E/J)\tau }_{{\mathrm{Signal}}}+\underbrace{\int_{0}^{\tau }[{\mathrm{d}}{W}_{1}(t)-{\mathrm{d}}{W}_{2}(t)]}_{{\mathrm{Noise}}}.$$

Considering the shot noises *W*_1,2_(*t*) of two measurements as uncorrelated and using the Wiener increment property ${\mathrm{d}}{W}_{1,2}^{2}(t)={\mathrm{d}}t$ we obtain SNR  = 2*γϵ*(Δ*E/J*)^2^*τ*. For a given averaging time *τ*, the condition SNR ≫1 provides us with the minimal energy difference we can distinguish $\Delta E/J\gg 1/\sqrt{2\gamma \epsilon \tau }$.

### Symmetries of the long-range transverse-field Ising model

The transverse-field Ising model (3) is invariant under the reflection and spin inversion symmetry transformations. We now provide an operational definition of these symmetries and the corresponding symmetry sectors.

Consider a product state vector in the *σ*^*x*^ basis $\left|\phi \right\rangle =\left|{s}_{1}^{x},\ldots, {s}_{N}^{x}\right\rangle$. The reflection operator can be defined by its action on the $\left|\phi \right\rangle$ state as $R\left|{s}_{1}^{x},\ldots ,{s}_{N}^{x}\right\rangle \equiv \left|{s}_{N}^{x},\ldots ,{s}_{1}^{x}\right\rangle$. Analogously, the spin inversion operator can be defined as $P\left|{s}_{1}^{x},\ldots, {s}_{N}^{x}\right\rangle \equiv \left|-{s}_{1}^{x},\ldots ,-{s}_{N}^{x}\right\rangle$. Both operators have two eigenvalues  ± and commute with each other and the Hamiltonian Eq. (3), thus representing QND observables, which can also be measured in the non-destructive way as presented in the paper.

The Hamiltonian can be independently diagonalized in each of the subspaces corresponding to eigenvalues of the *R* and *P* operators. The ground state of the Ising model with *J*, *h* > 0 belongs to the $\left\{+1,+1\right\}$ symmetry sector. For the test of ETH in Fig. 3 we consider the symmetry sector with eigenvalues of *R* and *P* given by $\left\{+1,-1\right\}$, respectively. The subspace can be reached from the $\left\{+1,+1\right\}$ sector by flipping odd number of spins (along the *σ*^*z*^ direction) in the limit of strong transverse field.

### Interaction renormalization

In numerical simulations in Fig. 3, we renormalize the interaction strength coefficient *J* such that the average interaction strength matches its value in thermodynamic limit. More precisely, for the *N*-spin Ising model (3) we rescale *J* → *J*_*N*_ ≡ *J* ⋅ *S*_*N*_ ∕ *S*_*∞*_, with ${S}_{N}\equiv \frac{1}{N}{\sum }_{i,j=1}^{N}1/{\left|i-j\right|}^{\alpha }$. The results are then expressed in units of *J*_14_.

### Work distribution function

The work distribution of a process defined by a time-dependent Hamiltonian $\hat{H}(t)$ (with the corresponding instantaneous energy eigenvalues and eigenstates ${E}_{\ell }^{t}$ and eigenstates ${\psi }_{\ell }^{t}$) is defined as follows^25^:

11 $${\mathcal{P}}(W)=\sum _{\ell {\ell }^{\prime}}\delta [W-({E}_{{\ell }^{\prime}}^{t}-{E}_{\ell }^{0})]{P}_{{\ell }^{\prime}\ell }^{t}{P}_{\ell }^{0},$$

where ${P}_{\ell }^{0}=\left\langle {\psi }_{\ell }^{0}\right|{\rho }_{{\rm{in}}}\left|{\psi }_{\ell }^{0}\right\rangle$ is the occupation probabilities of the initial state and ${P}_{{\ell }^{\prime}\ell }^{t}=\left| \left\langle {\psi }_{\ell^{\prime} }^{t}\left|U(t,0)\right|{\psi }_{\ell }^{0}\right\rangle \right|^{2}$ is the transition probabilities between initial *ℓ* and final ${\ell }^{\prime}$ states with $U(t,0)\equiv {\mathcal{T}}{e}^{-i{\int }_{0}^{t}\hat{H}({t}^{\prime}){\mathrm{d}}{t}^{\prime}}$ the evolution operator. The average of the exponentiated work in Eq. (7) is readily defined as an integral with the work distribution function Eq. (11): $\langle {e}^{-\beta W}\rangle \equiv \int {\mathrm{d}}W{e}^{-\beta W}{\mathcal{P}}(W)$.

## Supplementary information

Supplementary Information

## Data Availability

The data sets generated and analyzed in the current study are available from the corresponding author upon reasonable request.

## Code availability

The codes used to generate the numerical data in the current study are available from the corresponding author upon reasonable request.
