# Author Correction: Quantum non-demolition measurement of a many-body Hamiltonian

**DOI:** 10.1038/s41467-021-22105-3

**Published:** 2021-03-16

**Authors:** Dayou Yang, Andrey Grankin, Lukas M. Sieberer, Denis V. Vasilyev, Peter Zoller

**Affiliations:** 1 Center for Quantum Physics, University of Innsbruck, 6020 Innsbruck, Austria; 2 Institute for Quantum Optics and Quantum Information of the Austrian Academy of Sciences, 6020 Innsbruck, Austria

**Keywords:** Quantum simulation, Theoretical physics

Correction to: *Nature Communications* 10.1038/s41467-020-14489-5, published online 7 February 2020.

The original version of this Article omitted the following from the Acknowledgements: “FWF QuantERA via QTFLAG I03769”, which incorrectly read as “QuantERA via QTFLAG” in the original version. This has now been corrected in both the PDF and HTML versions of the Article.

