# Supplementary material for: Quantum non-demolition measurement of a many-body Hamiltonian
Source: Nat Commun. 2020 Feb 7;11:775. doi: 10.1038/s41467-020-14489-5 (PMC7005874; doi:10.1038/s41467-020-14489-5)
Supplement: Supplementary file 1 — Supplementary Information [file 41467_2020_14489_MOESM1_ESM.pdf]

Supplementary Information: Quantum Non-demolition Measurement of a Many-Body Hamiltonian

Dayou Yang et al.

This Supplementary Information is organized as follows: Supplementary Note I contains a detailed discussion of the implementation of the QND measurement scheme in a trapped-ion quantum simulator, including an analysis of the experimental feasibility of the scheme with different species of ions and using transverse or axial phonon modes. We provide additional information on applications of the QND scheme in Supplementary Note II and III. In particular, we give details on the numerical analysis of the ferromagnetic transition in the transverse-field Ising, and discuss prospects of an experimental test of the ETH.

Supplementary Note I: Implementation with trapped ions

In the main text we outline the implementation of our QND measurement scheme in a trapped-ion quantum simulator. In this section we elaborate on the detailed derivations behind the short presentation in the main text, and discuss the experimental feasibility of the proposed scheme. The section is structured as follows.

In Supplementary Note I A we introduce and provide an analytical study of the double Mølmer-Sørensen (MS) laser configuration (see Fig. 2 of the main text). We show that the low-frequency dynamics is governed by the effective system-meter coupling Hamiltonian $\hat{H}_{\mathcal{SM}}$, defined in Eq. (2) of the main text (we set $\hbar = 1$ hereafter)

$$\hat{H}_{\mathcal{SM}} = \hat{H}' \otimes \mathbb{I} + \vartheta \hat{H} \otimes \hat{P}, \tag{1}$$

where $\hat{P} \equiv i(\hat{a}_0^\dagger - \hat{a}_0)/\sqrt{2}$ is the quadrature operator of the center-of-mass (COM) phonon mode, with $\hat{a}_0(\hat{a}_0^\dagger)$ the corresponding annihilation(creation) operator. Both $\hat{H}$ and $\hat{H}'$ are many-body spin Hamiltonians of the Ising type,

$$\hat{H} = -\sum_{i<j}^{N} J_{ij}\hat{\sigma}_i^x\hat{\sigma}_j^x - h\sum_{j=1}^{N}\hat{\sigma}_j^z, \tag{2}$$

$$\hat{H}' = -\sum_{i<j}^{N} J_{ij}\hat{\sigma}_i^x\hat{\sigma}_j^x - (B-h)\sum_{j=1}^{N}\hat{\sigma}_j^z. \tag{3}$$

By adjusting the transverse field strength B, we are able to tune the measurement from QND ($B = 2h$) to imperfect QND ($B \simeq 2h$) which supports the observation of quantum jumps.

In Supplementary Note I B we provide a numerical study of the double MS scheme in different parameter regimes which supports the validity of the system-meter coupling Hamiltonian Eq. (1).

In Supplementary Note I C we describe the continuous readout of the spin Hamiltonian $\hat{H}$, achieved by sideband laser cooling of the motion of an ancilla ion at the edge of the ion chain and homodyne detection of its fluorescence, as schematically shown in Fig. 2(a) of the main text. We derive the resulting dynamics of the spin system as described by the stochastic master equation (SME)

$$d\hat{\rho}_c(t) = -i[\hat{H}', \hat{\rho}_c(t)]dt + \gamma\mathcal{D}[\hat{H}/J]\hat{\rho}_c(t)dt + \sqrt{\epsilon\gamma}\mathcal{H}[\hat{H}/J]\hat{\rho}_c(t)dW(t). \tag{4}$$

Here $\hat{\rho}_c(t)$ is the density matrix of the spin system conditioned on the homodyne detection signal, J is the characteristic energy scale of the spin Hamiltonian, γ is an effective measurement rate, ϵ is an overall detection efficiency and $dW(t)$ a white noise Wiener increment. For the detailed expressions of J and γ, cf. the main text or Supplementary Note I C below. The corresponding homodyne current reads

$$I(t) = 2\sqrt{\epsilon\gamma}\langle\hat{H}/J\rangle_c + \xi(t), \tag{5}$$

with $\xi(t)$ white (shot) noise $dW(t) \equiv \xi(t)dt$. We conclude Supplementary Note I C with a brief discussion on the filtering of the homodyne current.

In Supplementary Note I D we discuss some experimental considerations on the proposed trapped-ion implementation, including the analysis of its scalability, and the discussion of its robustness against major experimental imperfections. Supplementary Note I D also provides typical numbers for a proof-of-principle experiment.

We remark that our QND measurement scheme can be implemented with both transverse (x direction) and axial (z direction) phonon modes of the 1D ion string. While transverse phonon modes give rise to the power-law spin interactions $J_{ij} \propto |i-j|^{-\alpha}$ with $1 < \alpha < 3$ as is considered in the main text, axial phonon modes provide rich opportunities for engineering exotic spin couplings [1]. The derivation of our scheme for both cases is essentially the same. For notational concreteness, in the following Supplementary Notes I A and I C, we derive the equations by assuming transverse phonon modes. With the simple replacement $x \to z$ for the ionic motional operators, the same derivation applies to the axial case. In Supplementary Note I D, we discuss the features and experimental requirements of the transverse and the axial implementation separately.

A. Analytical study of the double Mølmer-Sørensen configuration

In this section we analyze in detail the laser configuration which generates the desired system-meter coupling $\hat{H}_{\mathcal{SM}}$ (1) as an effective Hamiltonian derived in perturbation theory for the laser assisted spin-mode couplings. While the model Hamiltonian in the main text refers to the lowest order terms in this expansion, we also derive the higher-order corrections to $\hat{H}_{\mathcal{SM}}$ and argue that they are indeed negligible under typical experimental conditions.

1. Light-ion coupling

We consider N ions trapped in a linear Paul trap. The internal structure of each ion is assumed to be a two level system (TLS), consisting of two qubit states $|\downarrow\rangle$ and $|\uparrow\rangle$. The transition $|\downarrow\rangle \rightarrow |\uparrow\rangle$ is driven by two pairs of laser beams, such that each pair realizes a Mølmer-Sørensen (MS) configuration, as shown schematically in Fig. 2 of the main text. The lasers which form the first pair, shown as the amber beams, are detuned by $\pm\Delta$ from the qubit transition frequency $\omega_{\uparrow\downarrow} \equiv E_\uparrow - E_\downarrow$ respectively, and have wave vector projections $\pm k$ along the x direction; The lasers corresponding to the second pair, shown as the blue beams, are detuned by $\pm\Delta'$ from $\omega_{\uparrow\downarrow}$, and have wave vector projections $\mp k$ along the x direction. In the frame rotating at $\omega_{\uparrow\downarrow}$, the full Hamiltonian of the internal and motional degrees of freedom (DOFs) of the ion chain reads

$$\hat{H}_{\text{full}} = \hat{H}_0 + \hat{V}. \tag{6}$$

Here $\hat{H}_0$ is the Hamiltonian of the external motion of the ions (along the x direction), and can be expressed in terms of the collective phonon modes

$$\hat{H}_0 = \sum_q \omega_q \hat{a}_q^\dagger \hat{a}_q. \tag{7}$$

where ω_q and $\hat{a}_q$ respectively denote the frequency and the annihilation operator of the mode q. Here, the modes are ordered according to their energy. For transverse phonon modes, the COM mode $q = 0$ has the highest frequency, and therefore $\omega_q > \omega_{q+1}$. The order of modes is reversed for axial phonon modes. The interaction between the ions and the lasers is described by the Hamiltonian

$$\begin{aligned}\hat{V} =&\frac{1}{2}\sum_{j=1}^{N}\hat{\sigma}_j^+\Big(\Omega_1 e^{-i\Delta t+ik\hat{X}_j+i\zeta_j^1}+\Omega_2 e^{i\Delta t-ik\hat{X}_j+i\zeta_j^2}\\&+\Omega_3 e^{-i\Delta' t-ik\hat{X}_j+i\zeta_j^3}+\Omega_4 e^{i\Delta' t+ik\hat{X}_j+i\zeta_j^4}\Big)+\text{H.c.}\end{aligned} \tag{8}$$

Here Ω_m with $m = 1, \ldots, 4$ denotes the Rabi frequency of the laser beams, which is assumed to be real and positive for concreteness. The lasers with indices $m = 1, 2$ correspond to the first MS pair and are shown as amber beams in Fig. 2 of the main text. The blue beams, which correspond to the lasers with indices $m = 3, 4$, form the second MS pair. For the j-th ion, $\hat{\sigma}_j^+ \equiv |\uparrow\rangle_j \langle\downarrow|$ is its internal raising operator, and ζ_j^m is the phase of laser m at its equilibrium position. The operator $\hat{X}_j$ describes the small-amplitude displacement from the equilibrium position along the x direction, and can be expressed in terms of the phonon operators as $k\hat{X}_j = \sum_q \eta_q M_{jq}\left(\hat{a}_q + \hat{a}_q^\dagger\right)$, where M_{jq} is the distribution matrix element of mode q, and the Lamb-Dicke (LD) parameters are defined as $\eta_q = \eta\sqrt{\omega_0/\omega_q}$, with $\eta = k/\sqrt{2m\omega_0}$.

Hereafter we consider the Rabi frequencies of the four laser beams being approximately equal up to a small offset,

$$\begin{aligned}\Omega_1 &= \Omega_3 = \Omega,\\ \Omega_2 &= \Omega_4 = \Omega + \delta\Omega.\end{aligned} \tag{9}$$

According to Supplementary Note I A 3, the small Rabi frequency mismatch $\delta\Omega$ creates the desired transverse field term of the Ising Hamiltonians (2) and (3), with the transverse field strength $h \propto \delta\Omega$.

A pair of Mølmer-Sørensen laser beams is known to create the Ising spin Hamiltonian Eq. (3) in the off-resonant regime $\Delta^{(\prime)} \gg \Omega$, $|\Delta^{(\prime)} - \omega_q| \gg \eta_q\Omega$ (see Refs. [2] and the discussion below). In our double MS configuration, however, an additional term describing the QND coupling between the Ising spin Hamiltonian and the COM phonon mode is generated [see the second term of Eq. (1)]. This is achieved by tuning $\Delta' = \Delta + \omega_0$, i.e., by choosing the beating between the two pairs of MS lasers to match the COM phonon excitation frequency. It leads to a resonant crosstalk between the two MS configurations, which results in the desired QND coupling term.

In the following, we derive Eq. (1) via a Magnus expansion of the time evolution of the ion chain in the interaction picture. We shall first introduce our method, which is a combined Magnus expansion and Lamb-Dicke expansion, in Supplementary Notes I A 2 and I A 3, respectively, and then work out the detailed expression of Eq. (1) order by order. We summarize the results in Supplementary Note I A 6.

2. Magnus expansion: effective Hamiltonian

Performing the gauge transformation $\hat{\sigma}_j^+ \rightarrow \hat{\sigma}_j^+ \exp\left[-i\left(\zeta_j^1 + \zeta_j^2\right)/2\right]$ and moving into the interaction picture with respect to $\hat{H}_0$, Eq. (8) becomes

$$\begin{aligned}\hat{V}_I =&\frac{\Omega}{2}\sum_{j=1}^{N}\hat{\sigma}_j^+\Big[e^{-i\Delta t+ik\hat{X}_j(t)+i\varphi_j}\\&+\left(1+\frac{\delta\Omega}{\Omega}\right)e^{i\Delta t-ik\hat{X}_j(t)-i\varphi_j}\\&+e^{-i\Delta' t-ik\hat{X}_j(t)+i(\theta+\varphi_j')}\\&+\left(1+\frac{\delta\Omega}{\Omega}\right)e^{i\Delta' t+ik\hat{X}_j(t)+i(\theta-\varphi_j')}\Big]+\text{H.c.}\end{aligned} \tag{10}$$

where the time-dependent position operator can be expressed in terms of the phonon modes as $k\hat{X}_j(t) = \sum_q \eta_q M_{jq}\hat{x}_q(t)$ with $\hat{x}_q(t) \equiv \hat{a}_q \exp(-i\omega_q t) + \text{H.c.}$, and the relative laser phases are denoted as $\theta = \left(\zeta_j^3 + \zeta_j^4 - \zeta_j^1 - \zeta_j^2\right)/2$, $\varphi_j = \left(\zeta_j^1 - \zeta_j^2\right)/2$, and $\varphi_j' = \left(\zeta_j^3 - \zeta_j^4\right)/2$. We note that the phase θ is independent of the ion index j. For the implementation with transverse phonons considered here, this is simply because the

laser phase ζ_j^m is independent of j. For the axial implementation, this is also true, as the position dependencies of the phases of two counter-propagating lasers cancel each other.

We consider the regime where the MS lasers drive the qubit transition and the phonon sidebands off-resonantly, $\Delta^{(\prime)} \gg \Omega$, $|\Delta^{(\prime)} - \omega_q| \gg \eta_q\Omega$. The evolution operator corresponding to Eq. (10) can be formally written as a Magnus series,

$$\hat{U}(t) \equiv \exp\left[-i\hat{G}(t)\right] = \mathcal{T}\exp\left[-i\int_0^t dt_1 \hat{V}_I(t_1)\right],$$
$$\hat{G}(t) = \sum_{l=1}^{\infty} \hat{G}_l(t). \tag{11}$$

Correspondingly, it allows us to define an effective Hamiltonian $\hat{H}_{\text{eff}} \equiv \lim_{t\to\infty} \hat{G}(t)/t$ which describes the slow dynamics of the ion chain on a time scale much longer than the phononic oscillation period $\sim 1/\omega_0$ [2]. The lowest-order terms of the Magnus series are given by

$$\hat{G}_1(t) = \int_0^t dt_1 \hat{V}_I(t_1)\, dt_1, \tag{12}$$
$$\hat{G}_2(t) = -\frac{i}{2}\int_0^t dt_1 \int_0^{t_1} dt_2 \left[\hat{V}_I(t_1), \hat{V}_I(t_2)\right], \tag{13}$$
$$\hat{G}_3(t) = -\frac{1}{6}\int_0^t dt_1 \int_0^{t_1} dt_2 \int_0^{t_2} dt_3 \left\{\left[\hat{V}_I(t_1), \left[\hat{V}_I(t_2), \hat{V}_I(t_3)\right]\right] + \left[\left[\hat{V}_I(t_1), \hat{V}_I(t_2)\right], \hat{V}_I(t_3)\right]\right\}. \tag{14}$$

In the next section we derive $\hat{H}_{\text{eff}}$ via explicit calculation of $\hat{G}(t)$ in the long-time limit. In this calculation we further perturbatively expand $\hat{V}_I$ in terms of the small Lamb-Dicke parameter $\eta \ll 1$. This allows us to construct $\hat{H}_{\text{eff}}$ order by order as a systematic expansion in η.

3. Expansion with respect to the Lamb-Dicke parameter η

We now construct the effective Hamiltonian $\hat{H}_{\text{eff}}$ as an expansion with respect to the Lamb-Dicke parameter, $\hat{H}_{\text{eff}} = \sum_{\ell=0}^{\infty} \hat{H}_{\text{eff}}^{(\ell)}$ with $\hat{H}_{\text{eff}}^{(\ell)} \propto \eta^\ell$. In order to do that, we first expand the interaction Hamiltonian Eq. (10) as $\hat{V}_I = \sum_{\ell=0}^{\infty} \hat{V}_I^{(\ell)}$, with

$$\hat{V}_I^{(\ell)}(t) = \frac{1}{2\times \ell!}\sum_{j=1}^{N} \hat{\sigma}_j^+ \left[i\sum_q \eta_q M_{jq}\hat{x}_q(t)\right]^\ell \times \left[\Omega e^{-i\Delta t + i\varphi_j} + (-1)^\ell (\Omega+\delta\Omega) e^{i\Delta t - i\varphi_j} + (-1)^\ell \Omega e^{-i\Delta' t + i(\theta+\varphi_j')} + (\Omega+\delta\Omega) e^{i\Delta' t + i(\theta-\varphi_j')}\right] + \text{H.c.} \tag{15}$$

To simplify the analysis, hereafter we consider $\theta = 0$. Moreover, we choose $\varphi_{j+1} - \varphi_j = 2\pi s$, $\varphi_{j+1}' - \varphi_j' = -2\pi s$ with $s \in \mathbb{Z}$. For the implementation using transverse phonon modes, this condition is automatically satisfied, with $s = 0$. For the implementation using axial phonon modes, this can be achieved by using the central part of an ion chain in a standard Paul trap with nearly uniform spacing d (or alternatively by using ions in equal-distance ion traps [3–6]) and by choosing an appropriate wavevector k of the MS beams such that $kd = 2\pi s$.

4. Contributions from $\hat{G}_1(t)$

Substituting the expression Eq. (15) into Eq. (13) and taking into account the conditions $|\Delta| \gg \Omega$ and $|\Delta - \omega_q| \gg \eta_q\Omega$, we immediately see that $\hat{G}_1(t)$ does not contribute to $\hat{H}_{\text{eff}}$. Indeed, $\hat{G}_1(t)$ describes small-amplitude fast oscillations at frequency $\sim \Delta$, which average to zero in the long-time regime.

5. Contributions from $\hat{G}_2(t)$ up to η^3: $\hat{H}_{\mathcal{SM}}$

Below, we derive the contribution from $\hat{G}_2(t)$ as an expansion in the Lamb-Dicke parameter. In this derivation, we implicitly assume that the small offset of the Rabi frequency [see Eq. (9)] satisfies $\delta\Omega/\Omega \sim O(\eta_q^2)$.

a. Transverse field terms.

The zeroth-order expansion of $\hat{H}_{\text{eff}}$ is readily constructed by plugging $\hat{V}_I^{(0)}$ into Eq. (13),

$$\hat{H}_{\text{eff}}^{(0)} = -\frac{i}{2t}\int_0^t dt_1 \int_0^{t_1} dt_2 \left[\hat{V}_I^{(0)}(t_1), \hat{V}_I^{(0)}(t_2)\right] = h\sum_{j=1}^{N} \hat{\sigma}_j^z. \tag{16}$$

We note that here and below, we implicitly assume the long-time limit $t \to \infty$. The zeroth-order contribution provides the transverse field term of the quantum Ising Hamiltonian, with the transverse field strength given by

$$h = \frac{\Omega\delta\Omega}{2}\left(\frac{1}{\Delta} + \frac{1}{\Delta'}\right). \tag{17}$$

Similarly, the first-order expansion $\hat{H}_{\text{eff}}^{(1)}$ is given by

$$\hat{H}_{\text{eff}}^{(1)} = -\frac{i}{2t}\sum_{\ell+m=1}\int_0^t dt_1 \int_0^{t_1} dt_2 \left[\hat{V}_I^{(\ell)}(t_1), \hat{V}_I^{(m)}(t_2)\right] = -\vartheta h \sum_{j=1}^{N} \hat{\sigma}_j^z \otimes \hat{P}, \tag{18}$$

where $\hat{P} \equiv i(\hat{a}_0 e^{i\varphi} - \hat{a}_0^\dagger e^{-i\varphi})/\sqrt{2}$ is a quadrature operator of the COM phonon mode, with $\varphi \equiv \varphi_j - \varphi_j'$ an angle

dependent on the laser phases, and $\vartheta = -\eta_0\sqrt{2}M_{i0} \simeq -\eta_0\sqrt{2/N}$ the dimensionless coupling strength. In the following, we absorb the phase φ into the definition of $\hat{a}_0$, $\hat{a}_0 e^{i\varphi} \to -\hat{a}_0$, thus $\hat{P} = i(\hat{a}_0^\dagger - \hat{a}_0)/\sqrt{2}$.

b. Ising terms.

The second order expansion of $\hat{H}_{\rm eff}$ can be constructed analogously,

$$\hat{H}_{\rm eff}^{(2)} = -\frac{i}{2t}\sum_{\ell+m=2}\int_0^t dt_1 \int_0^{t_1} dt_2 \left[\hat{V}_I^{(\ell)}(t_1), \hat{V}_I^{(m)}(t_2)\right] = -\sum_{i<j} J_{ij}\hat{\sigma}_i^x\hat{\sigma}_j^x. \tag{19}$$

In this derivation we dropped terms $\sim \eta^2\delta\Omega$ under our assumption $\delta\Omega/\Omega \propto \eta^2$. We discuss the effect of these higher-order terms in Supplementary Note I A 7. Equation (19) describes an Ising spin-spin coupling with coupling strength

$$J_{ij} = -\,\Omega^2 \sum_q \eta_q^2 \omega_q M_{iq} M_{jq} \times \left[\frac{1}{\Delta^2 - (\omega_q)^2} + \frac{1}{(\Delta')^2 - (\omega_q)^2}\right], \tag{20}$$

which includes two independent contributions from the two MS laser configuration.

Finally, the third order expansion of $\hat{H}_{\rm eff}$ can be calculated in an analogous (though lengthy) way

$$\hat{H}_{\rm eff}^{(3)} = -\frac{i}{2t}\sum_{\ell+m=3}\int_0^t dt_1 \int_0^{t_1} dt_2 \left[\hat{V}_I^{(\ell)}(t_1), \hat{V}_I^{(m)}(t_2)\right] = -\vartheta\left(\sum_{i<j} J_{ij}\hat{\sigma}_i^x\hat{\sigma}_j^x + \mathcal{E}\right) \otimes \hat{P}. \tag{21}$$

Here, the spin-spin interaction strength J_{ij} is defined in Eq. (20), and ϑ and $\hat{P}$ are defined below Eq. (18). $\mathcal{E} \equiv \sum_j J_{jj}/2$ is a constant driving field for the COM quadrature, which we neglect in the following as it just leads to a constant component in the measured signal. Equation (21) results from a resonant cross-talk between the two MS laser configuration under the condition $\Delta' = \Delta + \omega_z$, and describes the QND coupling between the spin Hamiltonian and the quadrature of the COM phonon mode.

In deriving Eq. (21), an important assumption we made is that no other phonon mode is resonantly excited except the COM mode up to third order in the Lamb-Dicke parameter. This "single sideband addressibility" is guaranteed by the condition

$$\sum_q \frac{\eta_q^2\Omega^2\omega_q\eta_p}{[\Delta^{(\prime)}]^2 - \omega_q^2} \ll |\omega_p - \omega_0|, \qquad \forall p \neq 0. \tag{22}$$

Equation (22) can be interpreted physically as follows: In our scheme, the excitation of sideband phonons is achieved by simultaneously flipping *two* spins. The strength for simutaneously flipping spin i and j is given by J_{ij} in Eq. (20). As a result, the sideband addressing strength is $\sim \eta\Omega^2\sum_q \eta_q^2\omega_q/\{[\Delta^{(\prime)}]^2 - \omega_q^2\}$ (we set $M_{iq} = 1$ for a worst-scenario analysis), and should be much smaller than the spectral gap between the COM mode and other modes.

In a transverse-phonon implementation, the phonon spectrum gets denser for increasing number of ions. Thus the validity of condition (22) sets a limit on the scalability of our QND scheme. This is analyzed in detail later in Supplementary Note I D. Here, we only note that Eq. (22) is much less stringent than the requirement for sideband addressing via laser flipping *individual* spins, $\eta_q\Omega \ll |\omega_q - \omega_0|, \quad \forall q \neq 0$, as is required, e.g., by the Cirac-Zoller gate or the near-resonant Mølmer-Sørensen gate. This leads to nice scalability of our QND measurement scheme for a given laser power.

Combining Eqs. (16), (18), (19) and (21), the effective Hamiltonian of the ion chain $\hat{H}_{\rm eff}$ can be written in the form of $\hat{H}_{\mathcal{SM}}$ in Eq. (1), with the identification

$$\hat{H} = -\sum_{i<j} J_{ij}\hat{\sigma}_i^x\hat{\sigma}_j^x - h\sum_{j=1}^N \hat{\sigma}_j^z, \qquad \hat{H}' = -\sum_{i<j} J_{ij}\hat{\sigma}_i^x\hat{\sigma}_j^x + h\sum_{j=1}^N \hat{\sigma}_j^z, \tag{23}$$

$\vartheta \simeq -\eta_0\sqrt{2/N}$ and $\hat{P} = i(\hat{a}_0^\dagger - \hat{a}_0)/\sqrt{2}$.

6. Tuning of $\hat{H}_{\mathcal{SM}}$

Here we describe a method to further tune the transverse field in $\hat{H}$ and $\hat{H}'$ [cf. Eq. (23)] independently, thus allowing to reach the QND sweetspot $\hat{H} = \hat{H}'$. To this end, we consider the same laser configuration as in Supplementary Note I A 1, nevertheless the detunings of the MS lasers are now respectively modified to $B \pm \Delta$, $B \pm \Delta'$, with $B \sim J \ll \Delta, \Delta'$. In the frame rotating at frequency $\omega_{\uparrow\downarrow} + B$, we get an additional term $B\sum_{j=1}^N \hat{\sigma}_j^z$ in the Hamiltonian of the laser-driven ion chain $\hat{H}_{\rm full}$ [cf. Eq. (6)]. Repeating the same derivation as described in Supplementary Notes I A 1 and I A 3, we recover exactly the same $\hat{H}$ that is coupled to the meter DOFs, while H' is modified as

$$H' = \sum_{i<j} J_{ij}\hat{\sigma}_i^x\hat{\sigma}_j^x - (h - B)\sum_j \hat{\sigma}_j^z.$$

By choosing $B = 2h$ we realize the QND condition $\hat{H}' = \hat{H}$, while offsetting B slightly from $2h$ allows us to observe quantum jumps between different eigenstates of $\hat{H}$.

7. Contributions from $\hat{G}_2(t)$ beyond η^3: higher-order corrections

In this section we derive the corrections to the QND Hamiltonian Eq. (1) resulting from higher-order terms in the Lamb-Dicke expansion of $\hat{G}_2(t)$. We show that these terms do not change the QND character of the proposed measurement scheme.

By straight forward calculation, we find that the fourth order expansion of the effective Hamiltonian can be written as

$$\begin{aligned}\hat{H}_{\text{eff}}^{(4)} &= -\frac{i}{2t}\sum_{\ell+m=4}\int_0^t dt_1\int_0^{t_1} dt_2\left[\hat{V}_I^{(\ell)}(t_1),\hat{V}_I^{(m)}(t_2)\right]\\ &= -\sum_{i<j} J_{ij}^{(4)}\hat{\sigma}_i^x\hat{\sigma}_j^x, \end{aligned}\tag{24}$$

with the spin-spin coupling

$$\begin{aligned}J_{ij}^{(4)} =& -\frac{\Omega^2}{2}\sum_{qp}\eta_q^2\eta_p^2 M_{iq}M_{jq}M_{ip}M_{jp}(\omega_q+\omega_p)\\ &\times\left[\frac{1}{\Delta^2-(\omega_q+\omega_p)^2}+\frac{1}{(\Delta')^2-(\omega_q+\omega_p)^2}\right]\\ &+\frac{\Omega^2}{2}\eta_0^2\omega_z\sum_p\eta_p^2(M_{ip}^2+M_{jp}^2)\\ &\times\sum_q M_{iq}M_{jq}\left[\frac{1}{\Delta^2-\omega_q^2}+\frac{1}{(\Delta')^2-\omega_q^2}\right].\end{aligned}\tag{25}$$

In the derivation of Eq. (24), we dropped terms proportional to the phonon-occupation under the assumption $\langle\hat{a}_q^\dagger\hat{a}_q\rangle \ll 1$.

Besides $\hat{H}_{\text{eff}}^{(4)}$, another correction to the QND Hamiltonian that is fourth order in η comes from the term $\sim \eta^2\delta\Omega \propto \eta^4$ which we dropped in Eq. (19). Via straightforward calculation we find this term can be written as a transverse field Ising Hamiltonian with site-dependent transverse field,

$$-\sum_{i<j} t_{ij}\hat{\sigma}_i^x\hat{\sigma}_j^x - \sum_j \lambda_j\hat{\sigma}_j^z,\tag{26}$$

with the coefficients

$$\begin{aligned}t_{ij} =& -\Omega\delta\Omega\sum_q \omega_q\eta_q^2 M_{iq}M_{jq}\\ &\times\left[\frac{1}{\Delta^2-\omega_q^2}+\frac{1}{(\Delta')^2-\omega_q^2}\right],\\ \lambda_j =& -2\Omega\delta\Omega\sum_q\eta_q^2 M_{jq}^2\left(\frac{1}{\Delta}+\frac{1}{\Delta'}\right).\end{aligned}\tag{27}$$

Importantly, the corrections to the QND Hamiltonian up to fourth order in the Lamb-Dicke parameter, Eq. (24) and (26), only involve spin DOFs and do not involve phonon DOFs. Thus, they only slightly renormalize the coefficients of $\hat{H}'$ [cf. Eq. (23)], introducing tiny mismatch between $\hat{H}'$ and $\hat{H}$. As described in the main text, these mismatch only introduce rare quantum jumps between energy eigenstates [cf. Fig. 1(3) of the main text], whereas the QND character of the measurement is maintained.

8. Effects of higher order Magnus series

In this section, we show that the contributions to the effective Hamiltonian from higher-order terms in the Magnus series, $\hat{G}_n(t), n \geq 3$, are of higher order than η^4. Thus they are much smaller than the system-meter coupling $\hat{H}_{\mathcal{SM}}$ and do not change the QND character of the proposed measurement scheme. The following analysis is based on power counting and physical arguments.

Let us first consider the contributions from $\hat{G}_3(t)$, cf. Eq. (14). For simplicity, we temporarily assume balanced MS configurations, i.e., $\delta\Omega = 0$. In this case, the spin operators $\hat{\sigma}_j^\pm$ in Eq. (15) can be combined to $\hat{\sigma}_j^x$. Therefore, spin operators always commute with each other at different time. As a result, the double commutator in the integrand of Eq. (14) is nonzero only if both the inner and outer commutators contain at least a pair of phonon annihilation and creation operators. Such an integrand, contains at least four phonon operators, and its contribution is $O(\eta^4)$ after the integration. Moreover, it is straightforward to see such a contribution contains a fast oscillating phase $\sim \Delta^{(\prime)}t$ and do not contribute to the effective Hamiltonian. Physically, it corresponds to processes which involve two virtual phonon excitations, which are nevertheless off-resonant. Thus, $\hat{G}_3(t)$ contributes $O(\eta^5)$ to the effective Hamiltonian when $\delta\Omega = 0$.

We now reintroduce the imbalance $\delta\Omega$. Keeping in mind that $\delta\Omega/\Omega \sim O(\eta^2)$, in the integrand of Eq. (15) we can restrict ourselves to 'relevant' terms that contain $\delta\Omega$ and at most two phonon operators, since the other terms contribute at $O(\eta^5)$ after multiplication with $\delta\Omega$. Under the conditions $\Delta^{(\prime)} \gg \Omega$ and $|\Delta^{(\prime)} - \omega_q| \gg \eta_q\Omega$, which are met in our off-resonant double MS configuration, it is straightforward to verify that the 'relevant' terms involving zero or one phonon operator contain fast oscillating phase $\sim \omega_z t$ and average to zero in the long time limit. The analysis of the terms consisting of $\delta\Omega$ and two phonon operators is more involved. First, we note that these terms are of the order $O(\eta^4)$, i.e., much smaller than the QND coupling Hamiltonian $\hat{H}_{\mathcal{SM}}$. Secondly, we find that they are off-resonant and average to zero if we avoid 'accidental' resonances, by requiring $|\omega_q - \omega_p| \neq |\Delta - \omega_0|, \forall p, q$, which can be achieved by choosing appropriate Δ in experiments. To summarize, the contribution of $\hat{G}_3(t)$ can be made as small as $O(\eta^5)$ and is thus negligible.

Next, we consider the contributions from $\hat{G}_4(t)$. Adopting similar arguments as above, we find that if $\delta\Omega = 0$ the contribution from $\hat{G}_4(t)$ is on the order of $O(\eta^6)$, and corresponds physically to processes involv-

ing three virtual phonon excitations. Reintroducing $\delta\Omega$ and looking at 'relevant' terms that contain at most two phonon operators, we find only one term that does not oscillate and remains finite in the long-time limit, under the assumption that accidental resonances are avoided and the phonon occupations are small, $\langle \hat{a}_q^\dagger \hat{a}_q \rangle \ll 1$. This term is proportional to $\delta\Omega \times \Omega^3/\Delta^3 \sum_j \hat{\sigma}_j^z$ and describes the AC-Stark shift from fourth-order perturbation theory. Comparing this term to the QND coupling terms (18) and (21), we find it has a relative strength $\Omega^2/(\eta\Delta^2)$. To ensure that this term has negligible effect on the performance of our QND scheme, we thus require $\Omega^2/(\eta\Delta^2) \ll 1$. This is typically true, as η and Ω/Δ are small parameters which have similar magnitudes.

Along the same lines, we can show that the contributions from $\hat{G}_n, n > 4$ are all negligible.

B. Numerical study of the double Mølmer-Sørensen configuration

We complement the analytical investigation of the double Mølmer-Sørensen laser configuration scheme above by a numerical study of the evolution of a system of $N = 3$ ions in a truncated phonon basis, and we identify parameter regimes in which a description of the system in terms of the effective Hamiltonian Eq. (1) is valid. We consider two scenarios: First, we study the evolution with $\delta\Omega = 0$, which corresponds to the Ising model with no transverse field $h = 0$, and we assume that the QND scheme is implemented with transverse phonon modes. Under these conditions, we show that in a wide range of detunings Δ the exact dynamics of the joint system consisting of spin and phonon degrees of freedom modes is well reproduced by the effective Hamiltonian Eq. (1). Second, for a fixed value of the detuning Δ, we show that the effective Hamiltonian remains valid for a range of values of the transverse field h. This analysis assumes the use of axial phonon modes and is based on Floquet theory.

1. *Dynamics of phonon modes*

The starting point of our consideration is the full Hamiltonian of the system Eq. (10). We focus on the transverse phonon modes and analyse the tunability of the detuning Δ which defines the spin-spin interaction parameter α as discussed. We assume all Rabi frequencies to be equal ($\delta\Omega = 0$), which corresponds to the Ising model with no transverse field Eq. (17). In this case the full Hamiltonian simplifies to:

$$\hat{V}_I = \sum_{j=1}^{N} \sigma_j^x \Big[\cos\Big(\Delta t - k\hat{X}_j(t)\Big) + \cos\Big(\Delta' t + k\hat{X}_j(t)\Big)\Big]. \tag{28}$$

This implies that for an initial product state in the σ^x basis there is no dynamics of spin variables, and only the phonon modes evolve in time. If they are initially prepared in their vacuum states then, according to the effective Hamiltonian given in Eqs. (1) and (2), at some final time t_f we expect $\langle \hat{x}_q \rangle \propto \sqrt{\langle a_q^\dagger a_q \rangle} \propto \delta_{q,0}(\sum J_{ij} s_i^x s_j^x) \cdot t_f$, where $\{s_i^x\}$ denote the initial spin configuration.

To check the validity of our model we compute the mean phonon number $\langle a_q^\dagger a_q \rangle$ using the full Hamiltonian Eq. (28) for the corresponding initial spin states as a function of the detuning Δ, while keeping $\Delta' = \Delta + \omega_0$. The result is shown in Supplementary Fig. 1(a). We observe the appearance of resonances at certain values of Δ, where the evolution does not correspond to the effective Hamiltonian Eq. (1). Partially this can be explained by the poles of the fourth order expansion term of the J_{ij} matrix Eq. (25). The rest of resonances have lower amplitude and higher frequency and we attribute them to the many-phonon resonant processes which correspond to the higher-than-fourth order η-expansion terms.

Remarkably, there exist wide regions of detuning Δ free of resonances provided that there is space between the higher order harmonics of the phononic eigenfrequencies $\omega_0 \gg |\omega_0 - \omega_q|, \forall q$. As shown in Supplementary Fig. 1(b) the system dynamics in these regions is well reproduced by the effective model Eqs. (1), (20) such that the COM mode population reflects the eigenenergies of the spin system.

In order to show that the scheme scales well with the number of ions we present in Supplementary Fig. 2(a,b) results for $N = 6$ ions interacting via 6 phonon modes. One can see that effective QND dynamics represents the exact results as expected.

The setup which is described in this section is also of interest as a first proof-of-principle experimental test of the proposed QND coupling of the spin model to the COM phonons $\vartheta\hat{H} \otimes \hat{P}$. Importantly, it does not require implementation of the full QND scheme with the continuous readout as the mean phonon-number measurement can be done in a multi-shot fashion.

2. *Floquet spectrum analysis*

Here we verify the effective QND dynamics for various transverse field values. In particular, we perform a numerical simulation of the periodically driven system of $N = 3$ ions interacting via 3 axial phonon modes according to the full Hamiltonian $\hat{H}_{\rm full}(t)$ given by Eq. (6). We choose commensurate detunings $\Delta = -7\omega_0$, $\Delta' = -6\omega_0$, such that the overall dynamics is periodic with frequency ω_0. Next, the operator of unitary evolution $\hat{U}(t) = \mathcal{T}\exp\left[-i\int_0^t dt_1 \hat{H}_{\rm full}(t_1)\right]$ is numerically evaluated for one period of the oscillation. The logarithm of eigenvalues of $\hat{U}(2\pi/\omega_0)$ provides $E_\ell^{\rm Floquet}$ the quasi energies of the effective Hamiltonian.

In Supplementary Fig. 3(a) we compare the Floquet quasi energies $E_\ell^{\rm Floquet}$ (blue dotted lines) with the spec-

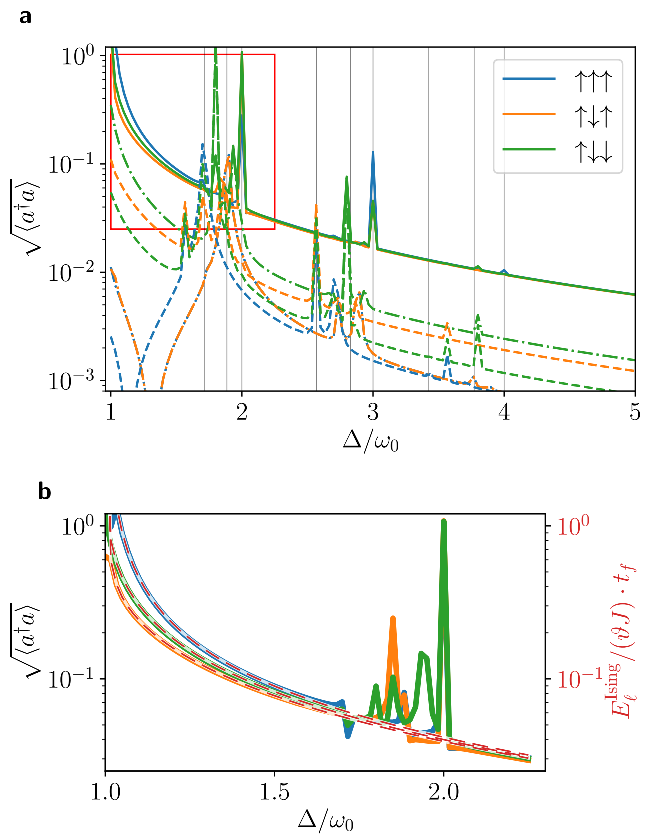

Supplementary Figure 1. (a) Transverse phonon mode occupation at the final time $t_f = 2\pi \times 600/\omega_0$ for different initial states of $N = 3$ spins indicated with blue, green, and orange colors. Solid, dot-dashed and dashed curves stand for the $q = 0, 1, 2$ modes, respectively. Vertical lines represent frequencies of the higher-order harmonics of the phonon modes. (b) Zoomed-in part [red rectangle in (a)] including the first resonances. Dashed curves show the analytical results obtained from Eqs. (1), (20). The numerical parameters: $\eta = 0.1$, $\Omega/\omega_0 = 1/6, \omega_0/\omega_z = 3$, the phonon modes are described by 5 Fock states.

trum $E_\ell^{\rm Ising}$ of the effective Ising Hamiltonian (3) with adjusted transverse field $B = 2h$ (dashed lines) for various values of the Rabi frequency mismatch $\delta\Omega$ expressed as a transverse field h via Eq. (17). The figure clearly shows that the exact eigenvalues $E_\ell^{\rm Floquet}$ are well represented by the effective Ising model.

Next, we study the coupling of the Ising Hamiltonian to the COM phonon mode. Here we consider the non-hermitian Hamiltonian of the full system (ions+phonons) $\hat{H}_{\rm full}(t) - i\frac{\gamma_s}{2} a_0^\dagger a_0$ with the non-hermitian term describing the decay of the COM mode due to the read-out. The Floquet eigenstates with the quasi energies around 0 and small imaginary parts represent the steady states of the open system. The COM mode displacements $\langle a_0 + a_0^\dagger \rangle$ averaged over these Floquet states are shown in Supplementary Fig. 3(b) with red lines. The displacement is proportional to the corresponding eigenenergy of the

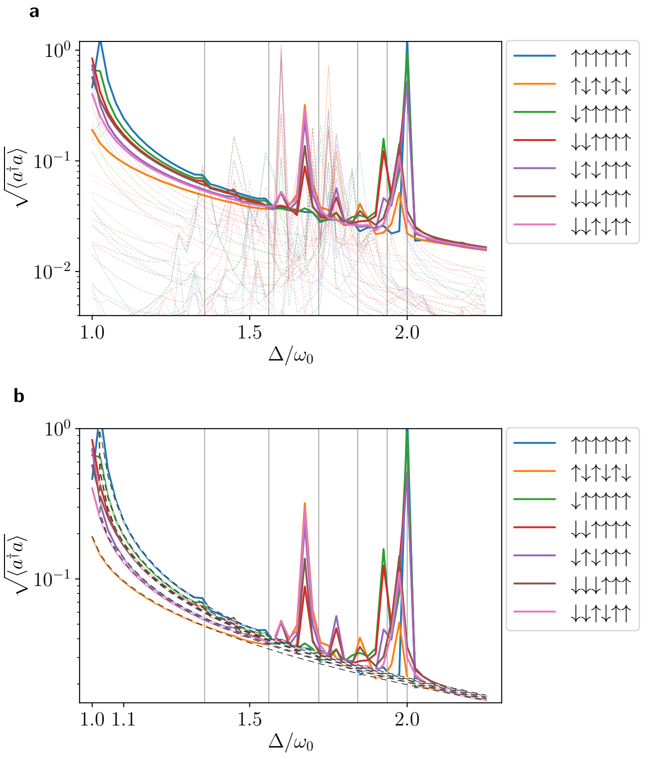

Supplementary Figure 2. (a) Transverse phonon mode occupation at the final time $t_f = 2\pi \times 400/\omega_0$ for different initial states of $N = 6$ spins indicated with various colors. Solid curves stand for the COM mode ($q = 0$) population, dashed curves represent the rest of the modes $q \neq 0$. Vertical lines represent frequencies of the higher-order harmonics of the phonon modes. (b) Comparison with the analytical results. Dashed curves show the analytical results obtained from Eqs. (1), (20). Parameters: $\eta = 0.1$, $\Omega/\omega_0 = 1/8, \omega_0/\omega_z = 4$. The phonon modes are described by 3 Fock states.

Ising Hamiltonian (2) shown with dashed lines. The resulting read-out photocurrent is sensitive to the amplitude of the COM mode oscillations and, therefore, reveals the eigenenergies of the desired Ising model.

C. Continuous readout of the spin Hamiltonian

With the implementation of the system-meter coupling Hamiltonian Eq. (1) at hand, in this section we present the detailed discussion on the readout of the transverse Ising Hamiltonian via continuous monitoring the center-of-mass phonon quadrature $\hat{X}$, extending the short description presented in the Method section of the main text.

The experimental setup we have in mind is shown schematically in Fig. 2 of the main text. Here, aside from the ions $j \in \{1, \dots, N\}$ which generates the QND Hamiltonian Eq. (1), an ancilla ion $j = 0$ is trapped at the edge of the ion chain and is subjected to sideband

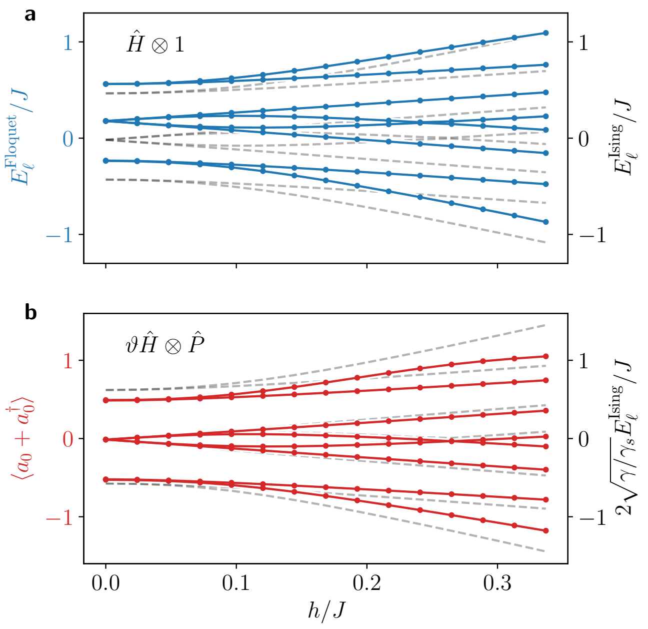

Supplementary Figure 3. Numerical verification of the double Mølmer-Sørensen configuration via Floquet analysis. (a) Test of the free evolution term $\hat{H}\otimes\mathbb{I}$ of the effective system-meter Hamiltonian (1). Blue lines show the exact Floquet spectrum depending on the effective transverse field h (see text), dashed lines represent eigenenergies of the effective Ising model. (b) The system-meter coupling $\vartheta\hat{H}\otimes\hat{P}$ test. Red lines show the COM mode displacement $\langle a_0+a_0^\dagger\rangle$ averaged over the exact Floquet eigenstates (see text), dashed lines represent the corresponding eigenenergies of the effective Ising model. The following parameters are used: $\eta=0.3$, $\Delta=-7\omega_0$, $\Delta'=-6\omega_0$, $\Omega=\omega_0$, $0<\frac{\delta\Omega}{\Omega}<2\times10^{-3}$, $\gamma_s=3\frac{\eta J}{\sqrt{N}}\left(\frac{\eta\Omega}{\Delta}\right)^2$. The COM mode is described by 6 Fock states, the other modes use 3 Fock states.

resolved laser cooling. The fluorescence emitted by the ancilla ion is collected by a lens setup and is continuously detected by a homodyne apparatus. We assume the MS lasers doesn't interact with the ancilla ion, nor does the cooling laser impact the ions $j\in\{1,\ldots,N\}$. As such, the ancilla ion participates in the collective vibrations of the ion chain and serves as a 'transducer' to couple light and phonons, thus allowing for monitoring the latter.

In the following, we introduce the quantum optical model for our considered setup in Supplementary Note IC1, using the language of a quantum stochastic Schrödinger equation (QSSE) (see, e.g., Chap. 9 in Ref. [7] for an introduction). Based on it, in Supplementary Note IC2 we derive a QSSE describing the coupling between the phonons and light by adiabatically eliminating the internal DOFs of the ancilla ion. Finally, in Supplementary Note IC3 we derive the stochastic master equation for continuous homodyne detection of the spontaneously emitted light and arrive at Eqs. (4) and (5).

1. *Quantum stochastic Schrödinger equation*

To be specific, we consider a standing-wave cooling configuration, i.e., the ancilla ion locates at the node of the standing wave [8]. In the interaction picture with respect to $\hat{H}_0$ [c.f. Eq. (7)], and in the frame rotating with the frequency of the cooling laser ω_L, the internal dynamics of the auxiliary ion is described by

$$\hat{H}_{\rm TLS}=-\Delta_e|e\rangle\langle e|+\frac{\Omega_0}{2}(|e\rangle\langle g|+{\rm H.c.})\sin[k_0\hat{X}_0(t)]. \quad (29)$$

Here, $|g\rangle(|e\rangle)$ is the ground(excited) level of the cooling transition respectively and $\Delta_e=\omega_L-\omega_{eg}$ is the frequency detuning between the cooling laser and the $|g\rangle\to|e\rangle$ transition. We assume the cooling laser is along the x axis, with wavevector k_0 and Rabi frequency Ω_0. The operator $\hat{X}_0(t)$ describes the (small-amplitude) displacement of the ancilla ion around its equilibrium position, and is related to the collective phonon modes of the ion chain by $\hat{X}_0(t)=\sum_q M_{0q}[\hat{a}_q(t)+\hat{a}_q^\dagger(t)]/\sqrt{2m_0\omega_q}$ with m_0 the mass of the ancilla ion, and $\hat{a}_q(t)=\hat{a}_q\exp(-i\omega_q t)$.

Besides the internal structure of the ancilla ion, the rest DOFs of our model includes the internal pseudo-spins of ion $j\in\{1,\ldots,N\}$ and the $N+1$ axial phonon modes. In the interaction picture with respect to $\hat{H}_0$, the time evolution of the total system is described by the (Itô) QSSE [7] for the ions and the external electromagnetic field (bath DOFs),

$$\begin{aligned}d|\Psi\rangle=&-i\left(\hat{H}_{\mathcal{SM}}+\hat{H}_{\rm TLS}-\frac{i}{2}\Gamma_e|e\rangle\langle e|\right)|\Psi\rangle dt\\&+\int du\sqrt{\Gamma_e N(u)}|g\rangle\langle e|e^{-ik_0u\hat{X}_0(t)}d\hat{B}^\dagger(u,t)|\Psi\rangle.\end{aligned} \quad (30)$$

In Eq. (30), the first line includes the spin-phonon Hamiltonian $\hat{H}_{\rm sys}$, the internal Hamiltonian of the ancilla ion $\hat{H}_{\rm TLS}$, and the spontaneous decay of the ancilla ion at a rate Γ_e. The second line describes spontaneous emission of the ancilla ion into the 3D electromagnetic modes. Here, the function $N(u)$ reflects the dipole emission pattern of the cooling transition, which, for the 1D ionic motion considered here, depends on a single variable $u\equiv\cos\nu\in[-1,1]$ with ν the angle between the wavevector of the emitted photon and the x axis. The spontaneous emission is accompanied by the momentum recoil described by the operator $e^{-ik_0u\hat{X}_0}$, with k_0 the wavevector of the emitted photon (approximately the same as the wavevector of the cooling laser). To account for the relevant electromagnetic modes in the emission direction u, quantum optics introduces the corresponding bosonic noise operators $\hat{b}_u(t)$ and $\hat{b}_u^\dagger(t)$, satisfying the white-noise commutation relations $[\hat{b}_u(t),\hat{b}_u^\dagger(t')]=\delta(u-u')\delta(t-t')$ [7]. In the Itô QSSE (30) these noise operators are transcribed as Wiener operator noise increments, $\hat{b}_u(t)dt\to d\hat{B}(u,t)$. Assuming the 3D bath is

initially in the vacuum state, they obey the Itô table [7],

$$\begin{aligned} d\hat{B}(u,t)d\hat{B}^\dagger(u',t) &= dt\delta(u-u'),\\ d\hat{B}^\dagger(u,t)d\hat{B}(u',t) &= 0,\\ d\hat{B}(u,t)d\hat{B}(u',t) &= d\hat{B}^\dagger(u,t)d\hat{B}^\dagger(u',t) = 0. \end{aligned} \tag{31}$$

We note, apart from the explicit ion-bath coupling in the second line of Eq. (30), the inclusion of the 3D electromagnetic field bath also introduces a decay term $-i\Gamma_e|e\rangle\langle e|/2$ in the first line of Eq. (30). Mathematically, this non-Hermitian term appears as an "Itô correction" when applying the Itô stochastic calculus to describe physical systems [7].

Based on Eq. (30), in the next section we derive a QSSE describing the coupling between the phonon modes and the electromagnetic field bath by adiabatically eliminating the internal dynamics of the ancilla ion.

2. *Adiabatic elimination of the internal dynamics of the ancilla ion*

We consider the following parameter regime. (i) The ancilla ion is weakly excited by the cooling laser, $\eta_q^0\Omega_0 \ll \Gamma_e$, where $\eta_q^0 \equiv k_0/\sqrt{2m_0\omega_q}$ is the Lamb-Dicke parameter corresponding to the cooling laser. (ii) The QND interaction is much weaker than the spontaneous emission strength of the ancilla ion, $|\hat{H}_{\mathcal{SM}}| \ll \Gamma_e$. (iii) The sideband resolved regime $\omega_q \gg \Gamma_e$. Condition (i) and (ii) guarantees that the internal dynamics of the ancilla ion is much faster than the dynamics of the rest of the system, allowing us to adiabatically eliminate the internal dynamics of the ancilla ion. Condition (iii) enables us to selectively enhance the center-of-mass phonon contribution in the detected photon current (see detailed discussion in Supplementary Note I C 3).

To perform the adiabatic elimination, we formally decompose the state of the total system [see Eq. (30)] into two components, $|\Psi\rangle = |\psi_e\rangle|e\rangle + |\psi_g\rangle|g\rangle$, with $|\psi_{e(g)}\rangle \equiv \langle e(g)|\Psi\rangle$. By the expansion up to second order in the small Lamb-Dicke parameter η_q^0, Eq. (30) becomes two coupled equations for $|\psi_{e(g)}\rangle$,

$$\begin{aligned} d|\psi_e\rangle =& -i\left[\hat{H}_{\mathcal{SM}} - \left(\Delta_e + \frac{i}{2}\Gamma_e\right)\right]|\psi_e\rangle dt\\ &- i\frac{\Omega_0}{2}\sum_q \eta_q^0 M_{0q}\left[\hat{a}_q^\dagger(t) + \hat{a}_q(t)\right]|\psi_g\rangle dt, \end{aligned} \tag{32}$$

$$\begin{aligned} d|\psi_g\rangle =& -i\hat{H}_{\mathcal{SM}}|\psi_g\rangle dt - i\frac{\Omega_0}{2}\sum_q \eta_q^0 M_{0q}\left[\hat{a}_q^\dagger(t) + \hat{a}_q(t)\right]|\psi_e\rangle dt\\ &+ \int du\sqrt{\Gamma_e N(u)}\Big\{1 - i\sum_q \eta_q^0 M_{0q}\left[\hat{a}_q^\dagger(t) + \hat{a}_q(t)\right]\\ &- \frac{1}{2}\Big[\sum_q \eta_q^0 M_{0q}\left[\hat{a}_q^\dagger(t) + \hat{a}_q(t)\right]\Big]^2\Big\} d\hat{B}^\dagger(u,t)|\psi_e\rangle. \end{aligned} \tag{33}$$

From Eq. (32) it is easy to see $|\psi_e\rangle \sim O(\eta_q^0)$. To keep $|\psi_g\rangle$ accurate to $O[(\eta_q^0)^2]$, we can neglect the second order Taylor expansion in the last term of Eq. (33).

Under conditions (i) and (ii) introduced in the beginning of this section, Eq. (32) can be solved adiabatically

$$|\psi_e\rangle = \frac{\Omega_0}{2}\sum_q \eta_q^0 M_{0q}\left[\frac{\hat{a}_q^\dagger(t)}{\Delta_e - \omega_q + \frac{i}{2}\Gamma_e} + \frac{\hat{a}_q(t)}{\Delta_e + \omega_q + \frac{i}{2}\Gamma_e}\right]|\psi_g\rangle.$$

Plugging the solution into Eq. (33), we arrive at a QSSE which describes the slow dynamics of the system assuming the ancilla ion staying in its internal stationary (ground) state,

$$\begin{aligned} d|\psi_g\rangle =& -i\left(\hat{H}_{\mathcal{SM}} + \sum_q \delta\omega_q \hat{a}_q^\dagger \hat{a}_q\right)|\psi_g\rangle dt\\ &- \frac{1}{2}\left(A_q^+ \hat{a}_q \hat{a}_q^\dagger + A_q^- \hat{a}_q^\dagger \hat{a}_q\right)|\psi_g\rangle dt\\ &+ \int du\sqrt{\Gamma_e N(u)}\hat{\mathcal{J}} d\hat{B}^\dagger(u,t)|\psi_g\rangle, \end{aligned} \tag{34}$$

where $dt \gg 1/\Gamma_e$ is the coarse-grained time increment and $d\hat{B}^\dagger(u,t)$ is the corresponding coarse-grained quantum noise increment. $\delta\omega_q$ is a (tiny) frequency renormalization of the q-th phonon mode,

$$\delta\omega_q = (\eta_q^0 M_{0q}\Omega_0)^2\left[\frac{\Delta_e + \omega_q}{4(\Delta_e+\omega_q)^2+\Gamma_e^2} + \frac{\Delta_e - \omega_q}{4(\Delta_e-\omega_q)^2+\Gamma_e^2}\right].$$

In the following we neglect such a tiny frequency shift. The damping rates $A_q^\pm$ for the q-th phonon mode are defined as

$$A_q^\pm = \frac{(\eta_q^0 M_{0q}\Omega_0)^2}{4(\Delta_e \mp \omega_q)^2 + \Gamma_e^2}\Gamma_e. \tag{35}$$

The operator $\hat{\mathcal{J}}$ is a collective quantum jump operator including all phonon modes,

$$\hat{\mathcal{J}} = \frac{\Omega_0}{2}\sum_q \eta_q^0 M_{0q}\left(\frac{\hat{a}_q^\dagger(t)}{\Delta_e - \omega_q + \frac{i}{2}\Gamma_e} + \frac{\hat{a}_q(t)}{\Delta_e + \omega_q + \frac{i}{2}\Gamma_e}\right). \tag{36}$$

The QSSE (34) describes the coupling between the phonon DOFs and the external electromagnetic field bath. This allows us to read out the COM quadrature $\hat{X}$ via homodyne detection of the external bath, as detailed in the next section.

3. *Homodyne detection of the fluorescence*

We consider continuous homodyne detection of the laser cooling fluorescence, as shown schematically in Fig. 1 of the main text. In such a measurement, the fluorescence photons are collected by linear optical elements, e.g., by a lens setup, and are then mixed with a

reference laser at a beam splitter. Photon counting of the mixed beam then allows for the measurement of the phase information of the fluorescence photons.

We assume the lens system covers a solid angle Ω, and define

$$\epsilon = \int_{\Omega} du N(u) \tag{37}$$

as the fraction of photons collected by the lens setup. The corresponding quantum noise increment is

$$d\hat{B}(t) = \frac{1}{\sqrt{\epsilon}} \int_{\Omega} du \sqrt{N(u)} d\hat{B}(u,t). \tag{38}$$

The homodyne measurement corresponds to making a measurement of the following quadrature operator [7, 9]

$$d\hat{Q}(t) = d\hat{B}(t)e^{-i\phi} + d\hat{B}^{\dagger}(t)e^{i\phi}, \tag{39}$$

with $\phi = (\omega_{\rm LO} - \omega_L)t + \phi_0$ and $\omega_{\rm LO}$ and ϕ_0 being the frequency and phase of the local oscillator. The measurement projects the state of the bath onto an eigenstate of $d\hat{Q}(t)$ corresponding to the eigenvalue $dq(t)$, which defines the homodyne current via $dq(t) \equiv I(t)dt$. It can be shown [7, 9] that the measurement outcome $dq(t)$ obeys a normal distribution centered at the mean value of the quantum jump operator $\hat{\mathcal{J}}$, i.e.,

$$dq(t) \equiv I(t)dt = \sqrt{\epsilon\Gamma_e}\langle \hat{\mathcal{J}}e^{-i\phi} + \hat{\mathcal{J}}^{\dagger}e^{i\phi}\rangle_c + dW(t), \tag{40}$$

where $dW(t)$ is a random Wiener increment, which is related to the shot noise by $dW(t) = \xi(t)dt$. The expectation value $\langle \ldots \rangle_c = {\rm Tr}(\ldots \mu_c)$ is taken with a conditional density matrix μ_c of the spin-phonon system. The evolution of μ_c is given by a SME derived from Eq. (34) by projecting out the bath DOFs following the standard procedure [7, 9],

$$\begin{aligned} d\mu_c =& -i[\hat{H}_{\mathcal{SM}}, \mu_c]dt + \sum_q \left(A_q^+ \mathcal{D}[\hat{a}_q^{\dagger}] + A_q^- \mathcal{D}[\hat{a}_q]\right)\mu_c dt \\ &+ \sqrt{\epsilon\Gamma_e}\mathcal{H}[\hat{\mathcal{J}}e^{-i\phi}]\mu_c dW(t), \end{aligned} \tag{41}$$

with $\mathcal{D}[\hat{O}]\rho \equiv \hat{O}\rho\hat{O}^{\dagger} - \frac{1}{2}\hat{O}^{\dagger}\hat{O}\rho - \frac{1}{2}\rho\hat{O}^{\dagger}\hat{O}$ being the Lindblad superoperator, and $\mathcal{H}[\hat{O}]\rho \equiv \hat{O}\rho - {\rm Tr}(\hat{O}\rho)\rho + {\rm H.c.}$ a superoperator corresponding to homodyne measurement. The first two lines of Eq. (41) is akin to the laser cooling master equation of trapped particles [7, 8], while the third line describes the measurement backaction of a continuous homodyne detection.

Under the condition of resolved sideband $\omega_q \gg \Gamma_e$, we can enhance the component corresponding to the COM phonon mode in the homodyne signal Eq. (40), by tuning the cooling laser in resonance with the red sideband of the COM mode, $\Delta_e = -\omega_0$. Under this condition, we have $\hat{\mathcal{J}} \simeq -i\Omega_0\eta_0^0 M_{00}\hat{a}_0 \exp(-i\omega_0 t)/\Gamma_e$ [see Eq. (36)], and $A_0^+ \simeq A_q^{\pm} \simeq 0$ for $q \neq 0$. Defining $\hat{\rho}_c^{\mathcal{SM}} = {\rm Tr}_{{\rm ph},q\neq 0}(\mu_c)$ by trancing out the phonon modes except for the COM mode, we have

$$\begin{aligned} I(t) =& \sqrt{2\epsilon\gamma_s}\langle \hat{X}\rangle_c + \xi(t), \\ d\rho_c^{\mathcal{SM}} =& -i[\hat{H}_{\mathcal{SM}}, \rho_c^{\mathcal{SM}}]dt + \gamma_s \mathcal{D}\left[\hat{a}_0\right]\rho_c^{\mathcal{SM}} dt \\ &+ \sqrt{\epsilon\gamma_s}\mathcal{H}\left[\hat{a}_0\right]\rho_c^{\mathcal{SM}} dW(t). \end{aligned} \tag{42}$$

where $\hat{X} = (\hat{a}_0 + \hat{a}_0^{\dagger})/\sqrt{2}$ is the x-quadrature of the COM phonon mode, $\gamma_s = (\Omega_0\eta_0^0 M_{00})^2/\Gamma_e$ is an effective measurement rate, with $M_{00} \simeq 1/\sqrt{N}$, and we choose $\omega_{\rm LO} - \omega_L = \omega_0$ and $\phi_0 = -\pi/2$ for the local oscillator to maximize the homodyne current.

Equation (42) already describes continuous QND readout of the transverse field Ising Hamiltonian. To simplify the analysis, we can further adiabatically eliminating the COM phonon mode in Eq. (42) under the condition $\gamma_s \gg \vartheta J$, and arrive at Eqs. (4) and (5) with the identification $\gamma \equiv 2J^2\vartheta^2/\gamma_s = 2\Gamma_e(\vartheta J/\Omega_0\eta_0^0 M_{00})^2$.

4. Filtering of the homodyne current

The homodyne current Eq. (5) is noisy, as it contains the (white) shot noise $\xi(t)$ inherited from the vacuum fluctuation of the electromagnetic field environment. To suppress the noise, we filter the homodyne current with a suitable linear lowpass filter

$$\mathcal{I}_{\tau}(t) = \int dt' h_{\tau}(t-t')I(t'), \tag{43}$$

where $h_{\tau}(t)$ is the filter function with a frequency bandwidth $\sim 1/\tau$, and $\mathcal{I}(t)$ is the *filtered homodyne current*. The filter attenuates the component of the shot noise with frequency higher than $1/\tau$ thus allowing us to extract out the signal we are interested in.

We adopt two filters in the main text. The first one is a simple *cumulative time-average*, $\overline{\mathcal{I}}(\tau) = (2N\sqrt{\gamma\epsilon}\tau)^{-1}\int_0^{\tau} dI(t)$. This allows us to attenuate the shot noise as much as possible, and is especially suitable for QND measurement (cf. Fig. 1e of the main text). In contrast, for imperfect QND measurement we are interested in resolving the quantum jumps between different energy eigenstates as a competition between coherent evolution and measurement backaction. To achieve this, we filter the homodyne current via $\mathcal{I}_{\tau}(t) = (2N\sqrt{\gamma\epsilon}\tau)^{-1}\int_0^{\infty} dt' e^{-t'/\tau} I(t-t')$ and call $\mathcal{I}_{\tau}(t)$ the *window-filtered homodyne current*. The time window τ is chosen to ensure $1/\gamma \ll \tau \ll T_{\rm dwell}$ with γ the measurement rate and $T_{\rm dwell}$ the typical time that the system dwells in particular eigenstates. This allows us to attenuate the shot noise as much as possible while still being able to resolve the quantum jumps.

D. Experimental feasibility

Having discussed our QND measurement scheme for the transverse-field Ising Hamiltonian in trapped-ion

setups, in this section, we show that state-of-the-art trapped-ion experiments provide all ingredients for the implementation of the QND scheme. First, in Supplementary Note I D 1, we summarize the experimental requirements of our scheme and discuss experimental imperfections including multiple sources of decoherence. We then discuss some practical points. These include the implementation of our scheme with axial and transverse phonon modes, analyzed in Supplementary Note I D 2 and Supplementary Note I D 3 respectively, as well as the implementation with different ion species, discussed in Supplementary Note I D 4. Finally, in Supplementary Note I D 5, we present experimental parameters for proof-of-principle realizations of our scheme.

1. *Experimental requirements and practical imperfections*

The performance of our QND measurement scheme depends on the collection efficiency ϵ of the photons scattered by the ancilla ion. A collection efficiency of 15 % is experimentally feasible for a single trapped ion [10], and we expect that a similar collection efficiency can be reached in our proposed setup. Even larger photon collection rates can be achieved by coupling the ancilla ion to optical cavities [11], or by simultaneous detection of the fluorescence of several ancilla ions.

In the implementation of homodyne detection of the spin system, we assume that the MS lasers do not interact with the ancilla ion, and that the cooling laser does not impact the ions $j \in \{1, \ldots, N\}$. These requirements can be met by individual addressing of each ion in realizing the MS configuration. Alternatively, this can be achieved by using global MS lasers and by choosing the ancilla ion from a different ion species [12, 13], so that the ancilla is decoupled from the MS lasers due to its different internal electronic structure. We note, however, that an ancilla ion with a different mass changes the structure of the COM mode. This has to be rectified in order to perform our QND measurement scheme, e.g., via local adjustments of the trapping potential near the ancilla ion using optical potentials [14].

Realistic trapped-ion systems have multiple sources of decoherence. The coherence time of current trapped-ion quantum simulators is limited by dephasing of the internal spins due to fluctuations of the global magnetic field which defines the quantization axis. Encoding the spins in ionic internal states which are first-order insensitive to magnetic field fluctuations greatly suppresses dephasing and extends the single-spin coherence time. This has been implemented, e.g., for $^{9}\mathrm{Be}^{+}$ ions (with a single-spin coherence time $t_{\mathrm{coh}} \sim 1.5\,\mathrm{s}$ [15]) and for $^{171}\mathrm{Yb}^{+}$ ions (with $t_{\mathrm{coh}} \sim 2.5\,\mathrm{s}$ [16]). Without this type of encoding, the coherence time is typically one order of magnitude shorter. For example, for $^{40}\mathrm{Ca}^{+}$ ions $t_{\mathrm{coh}} \sim 95\,\mathrm{ms}$ [17].

Another important source of decoherence is phonon heating due to electromagnetic field noise. In standard linear Paul traps, the phonon heating rate is typically below 1/s and is thus negligible [18]. Nevertheless, in surface ion traps, phonon heating is much more significant due to the short distance between the ions and the trap electrodes. Operating at cryogenic temperature can reduce phonon heating significantly. For example, the phonon heating rate of axial phonons is reduced to values as low as 70/s for ion spacings of $d \sim 30\,\mu\mathrm{m}$ in the cryogenic surface traps which are used by the NIST group [19]. Even lower phonon heating rates are being actively pursued [20].

In Supplementary Notes I D 4 and I D 5 further below, we show that the proposed QND measurement requires a time much shorter than the coherence time of trapped ions which is limited by the factors outlined above. Thus, current trapped-ion technology allows for robust implementation of our QND measurement scheme.

2. *Implementation with axial phonon modes*

In this section, we present some considerations on implementation of our QND measurement scheme with axial phonon modes.

The spectrum of axial phonon modes of an ion string in a linear Paul trap is extensive, i.e., it broadens with increasing number of ions N. To implement the long range Ising model with dipolar interaction $J_{ij} \propto 1/|i-j|^3$, the detunings $\Delta(\Delta')$ of the double MS configuration should also increase with the number of ions. Thus, to keep the spin-spin coupling $J \propto (\Omega/\Delta)^2(k^2/2m)$ [see Eq. (20)] finite, the power of the MS laser beams also goes up with increasing N. The achievable laser power in the laboratory thus puts a practical limitation on the scalability of the implementation wit axial phonon modes. On the other hand, the implementation with axial phonon modes benefits a relative large system-meter coupling $\hat{H}_{\mathcal{SM}}$, thanks to the large Lamb-Dicke parameter η associated with axial phonon modes (we note that in $\hat{H}_{\mathcal{SM}}$, $\vartheta \propto \eta$). In view of these, the implementation with axial phonon modes best serves as a small-scale proof-of-principle experiment, which demonstrates our proposed QND measurement and its applications. We provide the typical experimental parameters for such an implementation in Supplementary Note I D 5.

Moreover, we comment that the extensive feature of the axial phonon spectrum allows for engineering exotic spin coupling that goes beyond the power-law coupling, e.g., frustrated spin models, by carefully adjusting the laser detunings $\Delta^{(\prime)}$ with respect to the phonon spectrum [1]. The associated QND measurement could possibly enable rich opportunities for the study of these models and the preparation of their eigenstates.

3. *Implementation with transverse phonon modes*

Here we present some considerations concerning the implementation of our QND measurement scheme with

transverse phonon modes.

In contrast to axial phonon modes, the transverse phonon modes in a linear Paul trap have a dense spectrum of width $\propto \omega_z^2/\omega_x$, which is almost independent of the number of ions N; Here, $\omega_{z(x)}$ is the trapping frequency along the axial (transverse) direction, respectively. As a result, the long range-Ising model and the associated QND measurement can be implemented by a double MS configuration for which the detunings $\Delta^{(\prime)}$ and the Rabi frequency Ω can be held fixed upon increasing the number of ions. This leads to better scalability regarding the laser power as compared to the implementation with axial phonon modes.

The scalability of an implementation with transverse phonon modes is limited by the condition Eq. (22), since longer ion chain leads to denser phonon spectrum which eventually violates Eq. (22). To estimate an upper limit of the ion number N, we note that the LHS of Eq. (22) is much smaller than $\sum_q \eta_q^2\Omega^2\eta/|\Delta^{(\prime)}-\omega_0| \ll \sum_q \eta_q^2\Omega$, the latter "$\ll$" coming from our off-resonance condition $|\Delta^{(\prime)}-\omega_0| \gg \eta\Omega$. Thus, Eq. (22) is well satisfied as long as $\sum_q \eta_q^2\Omega \le |\omega_1-\omega_0|$. We can estimate $\sum_q \eta_q^2\Omega$ as $N\eta^2\Omega$ and $|\omega_1-\omega_0|$ as ω_z^2/ω_x, thus the above condition becomes $N\eta^2\Omega \le \omega_z^2/\omega_x$. Moreover, to prevent zig-zag transition of a linear ion chain we require $\omega_x/\omega_z \ge 0.73N^{0.86}$ [21]. Combining these two conditions we find $N \le [\omega_z/(\eta^2\Omega)]^{0.54}$. The latter quantity is typically around 100 in experiments. Thus, according to these estimates, the implementation with transverse phonon modes allows for scaling up to hundreds of ions.

4. *Implementation with different ion species*

We now discuss and compare the implementation with different ion species. As already mentioned in Supplementary Note I D 1, different ion species have different coupling strengths to the laser fields, and thus different energy scales of the targeted spin models and the associated QND measurements. Further, different ion species possess different coherence time. These parameters impact the performance of an implementation of our QND measurement scheme. As examples, we consider three ion species that are commonly used in current trapped-ion experiments: $^{171}Yb^+$, $^{40}Ca^+$ and $^9Be^+$.

A key parameter to quantify the performance of the proposed QND measurement is the signal-to-noise ratio (SNR) of a single measurement run, see the discussion in Methods of the main text. This can be equivalently expressed in terms of the (dimensionless) energy resolution $\Delta E/J \sim 1/\sqrt{2\gamma\epsilon\tau}$, for given measurement strength γ, photon collection efficiency ϵ and filtering time τ (which is the same as the measurement time). The energy resolution represents our ability to distinguish two adjacent energy levels from the documented data of a single measurement run, and should be compared to the (dimensionless) mean many-body level spacing, which can be estimated as $N/2^N$.

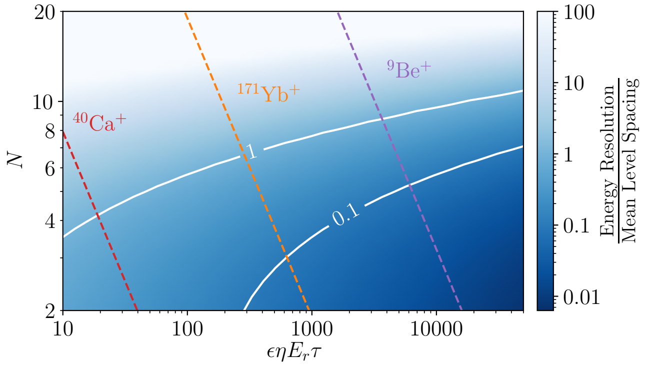

Supplementary Figure 4. Energy resolution of a single run of the QND measurement for different ions species. The (dimensionless) energy resolution $\Delta E/J$ over the mean level spacing $N/2^N$ is plotted against the ion number N and the dimensionless parameter $\epsilon\eta E_r\tau$, where ϵ is the detection efficency, η the Lamb-Dicke paramter E_r the recoil energy and τ the measurement time. The three dashed lines corresponds to estimations for three different ion species, for which we fix $\epsilon = 0.15, \eta = 0.1$ and choose $\tau = t_{\rm coh}/N$ with $t_{\rm coh}$ the single-spin coherence time.

The achievable measurement time τ can be estimated using the typical many-body dephasing time as $\tau = t_{\rm coh}/N$, with $t_{\rm coh}$ the single-spin coherence time and N the number of ions. The measurement strength γ of our QND scheme is controlled by the parameter $\vartheta J \sim (\eta/\sqrt{N})E_r(\Omega/\Delta)^2$, see the discussion in Supplementary Note I A. Here, E_r is the recoil energy of the qubit transition, whereas $\Omega/\Delta \ll 1$ is a small quantity independent of the ion species. As a result, we have the energy resolution $\Delta E/J \sim (\Delta/\Omega)N^{3/4}/\sqrt{2\eta\epsilon E_r t_{\rm coh}}$. Taking realistic experimental parameters, we find that the quantity $\eta\epsilon E_r t_{\rm coh}$ differs for different ion species and spans a range from 10^2 for $^{40}Ca^+$ to 10^4 for $^9Be^+$, see the vertical lines in Supplementary Fig. 4. Among the three ion species, $^9Be^+$ holds the promise of achieving the best energy resolution due to its light mass and long coherence time.

In Supplementary Fig. 4, we further plot the ratio between the energy resolution $\Delta E/J$ and the mean level spacing $N/2^N$, for increasing ion number N. Under current experimental conditions, the maximum system size N for which a single run of the QND measurement is able to resolve the eigenenergies can be estimated as $N \simeq 4$ for $^{40}Ca^+$, $N \simeq 6$ for $^{171}Yb^+$ and $N \simeq 8$ for $^9Be^+$. As a result, all three ion species are good candidates for building an intermediate-size interacting spin system for testing quantum fluctuation relations. The favorable scalability of $^9Be^+$ facilitates testing the eigenstate thermalization hypothesis. Finally, we note that these estimations concern a single measurement run and better energy resolution could be achieved by repeated measurements.

5. *Parameters for proof-of-principle experiments*

We proceed to present experimental parameters for a proof-of-principle implementation of our QND measurement scheme.

First, let us consider an implementation with ^{9}Be$^+$ ions and with axial phonon modes. The experimental system we have in mind is similar to the one reported in Ref. [22]. To be concrete, we consider $N = 5$ ^{9}Be$^+$ ions in a linear Paul trap. The internal spin of a ^{9}Be$^+$ ion consists of two hyperfine states driven by a Raman transition involving two 313 nm single-photon transitions with recoil energy $E_r = 2\pi \times 226.5\,$kHZ. We choose the axial trapping frequency $\omega_z = 2\pi \times 3\,$MHz, leading to a moderate Lamb-Dicke parameter of $\eta \simeq \sqrt{E_r/\omega_z} \simeq 0.27$. We further choose $\Delta = 3\omega_{q=4}$ and $\Omega = 0.15\Delta$ to stay in the off-resonant regime. The resulting spin-spin coupling strength is $J \simeq 2\pi \times 5\,$kHz, and the system-meter coupling is $\vartheta \simeq -0.17$. We choose the laser cooling rate of the ancilla ion $\gamma_s = 2\pi \times 5\,$kHz. Consequently, the effective measurement rate is $\gamma \simeq 2\pi \times 290\,$Hz. Assuming a photon collection efficiency $\epsilon = 0.15$ as discussed above, our QND measurement has a resulting characteristic time scale $1/\epsilon\gamma \simeq 3.7\,$ms, which is much shorter than the typical single qubit dephasing time $\sim 1\,$s [22]. Specifically, an averaging time $\tau = 10/\epsilon\gamma$ leads to an energy resolution (see Methods) $\Delta E/J \sim 0.22$, smaller than the minimal energy gap in this five-spin Ising model. This enables the preparation of single energy eigenstates via QND measurement, which suffices, e.g., for testing quantum fluctuation relations. This also allows for the observation of quantum jumps between different eigenstates in the imperfect QND regime as discussed in the main text.

Next, we provide experimental parameters for a transverse-phonon implementation realizing the power-law decaying spin-spin interactions, and discuss the associated energy resolution. These are relevant to the discussion in Supplementary Note III below on testing the eigenstate thermalization hypothesis. We consider $N = 6$ ^{9}Be$^+$ ions in a linear Paul trap with axial trapping frequency $\omega_z = 2\pi \times 2\,$MHz and transverse trapping frequency $\omega_x = 2\pi \times 8\,$MHz. We choose $\Omega = 2\pi \times 1.76\,$MHz, $\Delta = 2\pi \times 8.8\,$MHz, $\Delta' = \Delta + \omega_x$, and the Lamb-Dicke parameter along the transverse direction $\eta = 0.09 < \sqrt{E_r/\omega_x}$, which can be realized by properly choosing the direction of the double MS beams with respect to the ion string. The resulting spin-spin coupling strength obeys an approximate power law decay $J_{ij} \sim J/|i-j|^\alpha$ with $\alpha = 1.5$ and $J \simeq 2\pi \times 2.6\,$kHz. Further, this generate a QND coupling with strength $|\vartheta J| \simeq 2\pi \times 0.1\,$kHz. We choose the laser cooling rate of the ancilla ion $\gamma_s = 2\pi \times 0.5\,$kHz. Consequently, the effective measurement rate is $\gamma \simeq 2\pi \times 40\,$Hz. Assuming a photon collection efficiency $\epsilon = 0.15$, and a measurement time $\tau = 50\,$ms, the achieved energy resolution is $\Delta E/J \simeq 0.35$. This corresponds to a resolution of the energy density $\Delta\varepsilon = \Delta E/(JN) = 0.06$, which is indicated as horizontal error bars in Supplementary Fig. 8.

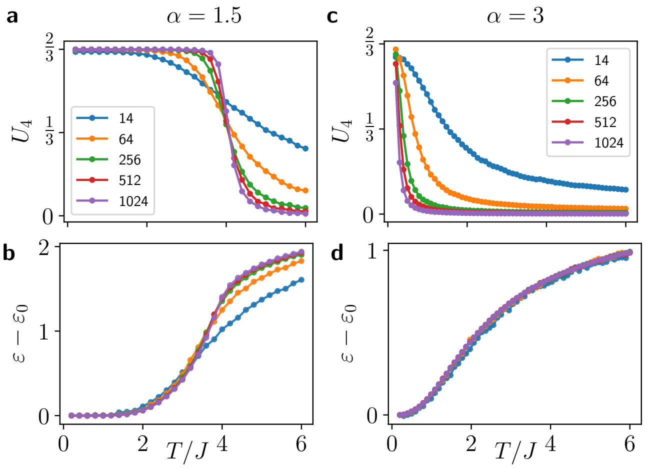

Supplementary Figure 5. Phase transition of the Ising model in the canonical ensemble. (a), (c) Binder cumulant as a function of temperature for different system sizes. (b), (d) the corresponding energy of the system.

Supplementary Note II: Numerical study of thermal properties of energy eigenstates

In this section we provide additional details on the numerical simulations used in our study of thermal properties of the energy eigenstates in the main text.

In Supplementary Note II A we describe the canonical-ensemble quantum Monte-Carlo simulations of the transverse-field Ising model used in Fig. 3 of the main text. We discuss the phase transition in the case of of long- and short-range interactions.

In Supplementary Note II B we discuss the phase diagram of the transverse field Ising model for realistic spin-spin interaction J_{ij} and show that it qualitatively agree with the one obtained using an approximate power-law.

A. Monte-Carlo simulations

Here we provide details on the numerical simulations of the phase transition of the Ising model in canonical ensemble $\hat{\rho}_{\text{th}}(T) \equiv e^{-\hat{H}/T}/\text{Tr}\left[e^{-\hat{H}/T}\right]$ using the quantum Monte-Carlo technique. It allows us to calculate the critical energy ε using the finite-size scaling analysis of the Binder cumulant, defined as

$$U_4 \equiv 1 - \frac{\langle \hat{m}_x^4 \rangle}{3\,\langle \hat{m}_x^2 \rangle^2}$$

By its construction [23], this cumulant distinguishes the ordered phase with $U_4 \approx 2/3$, from the disordered phase with $U_4 \approx 0$. As a result, when crossing the phase transition, the Binder cumulant has a sharp jump between

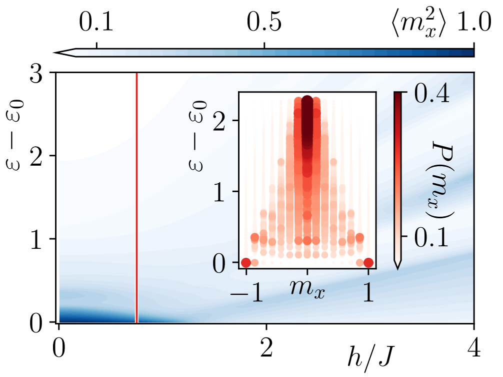

Supplementary Figure 6. Excited-state phase transition in the Ising model with $\alpha = 3$. (a) Ferro-paramagnet crossover in the Ising model of $N = 14$ spins prepared by the energy measurements in microcanonical ensembles of width $\Delta E/(JN) = 0.1$. The transition between magnetically ordered phase $\langle \hat{m}_x^2 \rangle_{\mathrm{mc}} \approx 1$ (dark blue) to disordered phase $\langle \hat{m}_x^2 \rangle_{\mathrm{mc}} \approx 0$ (light blue) is shown as function of the mean energy density $\varepsilon = \langle \hat{H} \rangle_{\mathrm{mc}} /(JN)$ and the transverse field h. Test of ETH for the symmetry sector $\{+1, +1\}$ is shown in the inset: only the ground state has a bimodal distribution $P(m_x)$.

these two values at the critical temperature T_c. This allows us to determine T_c for the Ising model. The results of the Monte-Carlo simulation for $\alpha = 1.5$, $h/J = 1$ and different system sizes N are shown in Supplementary Fig. 5(a). For a sufficiently large number of spins the curves of U_4 cross approximately at the same temperature, which provides a good estimate of T_c. The corresponding critical energy density $\varepsilon \equiv \mathrm{Tr}\left[\hat{H}\hat{\rho}_{\mathrm{th}}(T_c)\right]/NJ$ can be easily determined from the energy-temperature conversion curve shown in Supplementary Fig. 5(b).

We also study the Ising model with $\alpha = 3$ shown in Supplementary Fig. 5(c) and (d). The Binder cumulant curves show no crossing at finite temperature, which indicates the absence of thermal phase transitions as it should be in case of short-range interactions $\alpha > 2$ [24]. Below, we study if the same thermodynamic properties are exhibited by the individual eigenstates as can be expected if the ETH holds in this regime.

B. Realistic spin-spin interaction

For a finite ion chain, the spin-spin coupling J_{ij} Eq. (20) only approximately satisfy the power law. Here we study the phase diagram of the Ising model using these realistic spin-spin interaction coefficients J_{ij}, and show that it agrees well with the phase diagram of the power-law interaction model studied in the main text. We consider 14 ions in a linear Paul trap and use the transverse phonons to implement our QND measurement. We choose $\omega_x/\omega_z = 10$, $\Delta/\omega_z = 10.22$ and $\Delta'/\omega_z = 20.22$. By numerically calculate the phonon

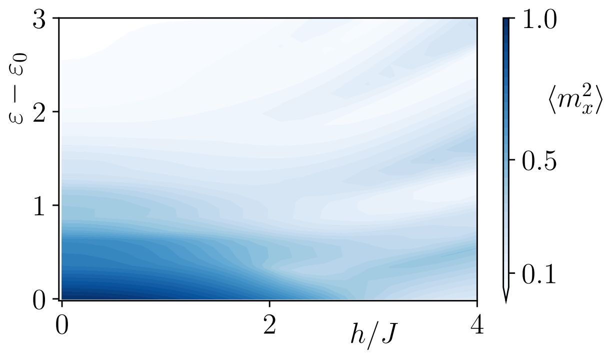

Supplementary Figure 7. Phase diagram of the transverse field Ising model $N = 14$ with the interaction coefficients J_{ij} computed according to Eq. (20). All the qualitative features are in good agreement with those shown in Fig. 3 (a) of the main text.

modes, we find that the spin-spin couplings in Eq. (8) of the main text satisfy approximately power-law decay, with the exponent $\alpha \simeq 1.5$ from a least-square fit. Based on these realistic spin-spin couplings, we calculate the phase diagram of the system as is shown in Supplementary Fig. 7. As can be seen all the features of the phase diagram are in good agreement with those shown in the main text, where we approximated $J_{ij} \propto 1/|i-j|^{1.5}$.

C. Case of short-range interactions

We now study the phase transition for the case $\alpha = 3$ in the microcanonical ensemble and on the level of individual eigenstates. The phase diagram in the microcanonical ensemble is shown in Supplementary Fig. 6. It is clearly visible that contrary to the long-range Ising model, the ordering remains only in the vicinity of $\epsilon \approx \epsilon_0$. This is also reflected by the order parameter probability distribution $P(m_x)$ for the individual eigenstates shown in inset of Supplementary Fig. 6, which shows bimodal behavior only for the ground state. We note that this observation is compatible with the eigenstate thermalization hypothesis.

Supplementary Note III: Eigenstate thermalization hypothesis

As explained in the main text, the ETH asserts a specific form of matrix elements of few-body observables in the energy eigenbasis of an ergodic many-body Hamiltonian [25]:

$$\langle \ell' | \hat{O} | \ell \rangle = O(\bar{E})\delta_{\ell'\ell} + e^{-S(\bar{E})/2} f_{\hat{O}}(\bar{E}, \omega) R_{\ell'\ell}. \quad (44)$$

In particular, diagonal and off-diagonal matrix elements are determined by functions $O(\bar{E})$ and $f_{\hat{O}}(\bar{E}, \omega)$, respectively, which depend smoothly on their arguments

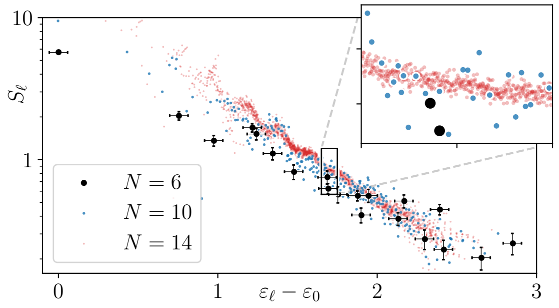

Supplementary Figure 8. Structure factor for single energy eigenstates for $N = 6, 10, 14$ (black, blue, red dots). The narrowing of fluctuations of the eigenstate expectation values with increasing system size is a clear indication of the occurrence of eigenstate thermalization. The Ising model parameters are $\alpha = 1.5$ and $h/J = 0.75$. The horizontal error bars indicate the estimated energy resolution for 6 spins. The vertical error bars provide an estimate of the result dispersion for 100 simulated experimental runs per eigenvalue.

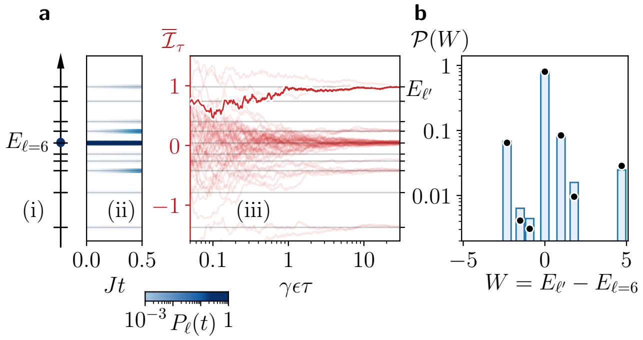

Supplementary Figure 9. Measuring off-diagonal matrix elements of a local observable $\hat{V} = \tilde{h}\,\hat{\sigma}_j^z$ in the energy eigenbasis via the work probability distribution $P(W)$. (a) Protocol to measure the work distribution $P(W)$ as described in the text. (i) The system of $N = 5$ spins is prepared in an energy eigenstate $|\ell = 6\rangle$ with energy $E_{\ell=6}$. (ii) By applying the perturbation $\hat{V}$ at the middle spin ($j = 3$ and $\tilde{h} = J$) during a time $J\Delta t = 0.5$, the system is driven into a superposition of eigenstates $|\ell\rangle$ with probabilities $P_\ell(t)$ (blue shading). (iii) A second measurement of energy collapses the state of the system continuously to a final state $|\ell'\rangle$. An exemplary trajectory is shown in dark red. Repeating steps (i), (ii), and (iii) produces a sample of trajectories (light red), which gives access to the full distribution $P(W)$ of work $W = E_{\ell'} - E_\ell$. (b) Normalized work distribution $\mathcal{P}(W) = P(W)/\sum_W P(W)$ (blue columns). For weak perturbations, the work distribution is determined by off-diagonal matrix elements $\langle\ell'|\hat{V}|\ell\rangle$ of the perturbation. The corresponding approximation to $\mathcal{P}(W)$ is indicated by black dots.

$\bar{E} = (E_\ell + E_{\ell'})/2$ and $\omega = E_{\ell'} - E_\ell$. $S(\bar{E})$ is the thermodynamic entropy at the mean energy $\bar{E}$, and $R_{\ell'\ell}$ is a random number with zero mean and unit variance. From the above form of matrix elements it follows that single energy eigenstates $|\ell\rangle$ encode thermodynamic properties such as phases and phase transitions which we typically associate with a microcanonical or canonical ensemble describing systems in thermodynamic equilibrium. In the main text, we describe how qualitative aspects of eigenstate thermalization can be probed across the ferromagnetic phase transition of the long-range transverse Ising model at finite energy density. Here, we elaborate on direct and more quantitative experimental tests for diagonal and off-diagonal matrix elements.

A. Diagonal matrix elements

Assessing ETH quantitatively requires to show that fluctuations of single-eigenstate expectation values $\langle\ell|\hat{O}|\ell\rangle$ around the microcanonical average $\mathrm{tr}(\hat{O}\hat{\rho}^{\mathrm{mc}}_{E_\ell})$ are suppressed with increasing system size [26]. Suitable expectation values for this purpose are fluctuations of the magnetization, $\langle\ell|\hat{m}_x^2|\ell\rangle$ where $\hat{m}_x = N^{-1}\sum_j \hat{\sigma}_j^x$, and the structure factor, $S_\ell \equiv N\langle\ell|\hat{m}_x^2|\ell\rangle$, which remain finite in the thermodynamic limit in the ordered and disordered phase, respectively. Using these quantities, numerical tests of ETH have been performed for the two-dimensional transverse Ising model with nearest-neighbor interactions [27], and in the one-dimensional model [28]. In experiments with the trapped ion toolbox, the system sizes for which single eigenstates can be prepared are limited by the increasing measurement time which is required to resolve many-body energy level splittings. Since for intermediate system sizes the number of states in the disordered phase exceeds the one in the ordered phase (see Fig. 3 in the main text), the most promising prospect to test ETH quantitatively in experiments is to consider the structure factor S_ℓ in the disordered phase. We demonstrate the narrowing with system size of state-to-state fluctuations of the structure factor for the one-dimensional transverse Ising model numerically in Supplementary Fig. 8. Even for 6 spins, for which we expect experiments with current technology to be able to resolve individual energy eigenstates as discussed in Supplementary Note I D 5 above, the structure factor exhibits relatively small state-to-state fluctuations, consistent with ETH. In the figure, we also indicate the estimated energy resolution for 6 spins, and the expected error in determining the structure factor from 1000 measurements. A further suppression of state-to-state fluctuations is clearly visible for 10 and 14 spin systems, which, however, require improved experimental coherence times and detection efficiencies.

B. Off-diagonal matrix elements

Off-diagonal matrix elements are encoded in the dynamics, e.g. in transition probabilities between energy

eigenstates in response to a weak perturbation. Using the trapped-ion QND toolbox, these transition probabilities are accessible through the protocol, which is illustrated in Supplementary Fig. 9(a): The preparation (i) of an eigenstate of $\hat{H}$ with energy E_ℓ at a given value h of the transverse field is followed by a period (ii) of length Δt of free evolution [$\vartheta = 0$ in Eq. (1)] according to a perturbed Hamiltonian $\hat{H}' = \hat{H} + \hat{V}$; this is followed by another measurement (iii) of $\hat{H}$ which yields a value $E_{\ell'}$. The measurement outcomes determine the work $W = E_{\ell'} - E_\ell$ performed on the system by the perturbation $\hat{V}$. To the lowest order in the perturbation, the work distribution is determined by off-diagonal matrix elements $\langle \ell' | \hat{V} | \ell \rangle$ in the energy eigenbasis, $P(W) = \delta_{\ell' \ell} + (\Delta t)^2 \, |\langle \ell' | \hat{V} | \ell \rangle|^2$. For $|\langle \ell' | \hat{V} | \ell \rangle|, W \ll (\Delta t)^{-1}$, we find good agreement between the exact work distribution and the lowest-order approximation as illustrated in Supplementary Fig. 9(b).

[1] Porras, D. & Cirac, J. I. Effective Quantum Spin Systems with Trapped Ions. *Phys. Rev. Lett.* **92**, 207901 (2004).

[2] Kim, K. *et al.* Entanglement and tunable spin-spin couplings between trapped ions using multiple transverse modes. *Phys. Rev. Lett.* **103**, 120502 (2009).

[3] Schulz, S. A., Poschinger, U., Ziesel, F. & Schmidt-Kaler, F. Sideband cooling and coherent dynamics in a microchip multi-segmented ion trap. *New Journal of Physics* **10**, 045007 (2008).

[4] Harlander, M., Lechner, R., Brownnutt, M., Blatt, R. & Hänsel, W. Trapped-ion antennae for the transmission of quantum information. *Nature* **471**, 200–203 (2011).

[5] Mehta, K. K. *et al.* Ion traps fabricated in a cmos foundry. *Applied Physics Letters* **105** (2014).

[6] Wilson, A. C. *et al.* Tunable spin–spin interactions and entanglement of ions in separate potential wells. *Nature* **512**, 57–60 (2014).

[7] Gardiner, C. & Zoller, P. *The Quantum World of Ultra-Cold Atoms and Light Book II* (ICP, London, 2015).

[8] Cirac, J. I., Blatt, R., Zoller, P. & Phillips, W. D. Laser cooling of trapped ions in a standing wave. *Phys. Rev. A* **46**, 2668–2681 (1992).

[9] Wiseman, H. M. & Milburn, G. J. *Quantum measurement and control* (CUP, Cambridge, 2009).

[10] Bushev, P. *et al.* Feedback cooling of a single trapped ion. *Phys. Rev. Lett.* **96**, 043003 (2006).

[11] Stute, A. *et al.* Toward an ion–photon quantum interface in an optical cavity. *Applied Physics B* **107**, 1145–1157 (2012).

[12] Tan, T. R. *et al.* Multi-element logic gates for trapped-ion qubits. *Nature* **528**, 380 (2015).

[13] Negnevitsky, V. *et al.* Repeated multi-qubit readout and feedback with a mixed-species trapped-ion register. *Nature* **563**, 527–531 (2018).

[14] Schneider, C., Enderlein, M., Huber, T. & Schaetz, T. Optical trapping of an ion. *Nature Photonics* **4**, 772 (2010).

[15] Langer, C. *et al.* Long-lived qubit memory using atomic ions. *Phys. Rev. Lett.* **95**, 060502 (2005).

[16] Olmschenk, S. *et al.* Manipulation and detection of a trapped yb$^+$ hyperfine qubit. *Phys. Rev. A* **76**, 052314 (2007).

[17] Monz, T. *et al.* 14-qubit entanglement: Creation and coherence. *Phys. Rev. Lett.* **106**, 130506 (2011).

[18] Roos, C. *et al.* Quantum state engineering on an optical transition and decoherence in a paul trap. *Phys. Rev. Lett.* **83**, 4713–4716 (1999).

[19] Brown, K. R. *et al.* Coupled quantized mechanical oscillators. *Nature* **471**, 196–199 (2011).

[20] McConnell, R., Bruzewicz, C., Chiaverini, J. & Sage, J. Reduction of trapped-ion anomalous heating by in situ surface plasma cleaning. *Phys. Rev. A* **92**, 1–5 (2015).

[21] Wineland, D. *et al.* Experimental issues in coherent quantum-state manipulation of trapped atomic ions. *Journal of Research of the National Institute of Standards and Technology* **103**, 259 (1998).

[22] Gaebler, J. P. *et al.* High-fidelity universal gate set for ^{9}Be$^+$ ion qubits. *Phys. Rev. Lett.* **117**, 060505 (2016).

[23] Binder, K. Critical properties from monte carlo coarse graining and renormalization. *Phys. Rev. Lett.* **47**, 693–696 (1981).

[24] Dutta, A. & Bhattacharjee, J. K. Phase transitions in the quantum ising and rotor models with a long-range interaction. *Phys. Rev. B* **64**, 184106 (2001).

[25] Srednicki, M. The approach to thermal equilibrium in quantized chaotic systems. *J. Phys. A. Math. Gen.* **32**, 1163–1175 (1999).

[26] D'Alessio, L., Kafri, Y., Polkovnikov, A. & Rigol, M. From quantum chaos and eigenstate thermalization to statistical mechanics and thermodynamics. *Adv. Phys.* **65**, 239–362 (2016).

[27] Mondaini, R., Fratus, K. R., Srednicki, M. & Rigol, M. Eigenstate thermalization in the two-dimensional transverse field Ising model. *Phys. Rev. E* **93**, 032104 (2016).

[28] Fratus, K. R. & Srednicki, M. Eigenstate Thermalization and Spontaneous Symmetry Breaking in the One-Dimensional Transverse-Field Ising Model with Power-Law Interactions. Preprint at http://arxiv.org/abs/1611.03992 (2016).
